# Spectral Tuning of Hyperbolic Shear Polaritons in Monoclinic Gallium Oxide via Isotopic Substitution

**DOI:** 10.1002/adma.202514561

**Published:** 2026-01-10

**Authors:** Giulia Carini, Mohit Pradhan, Elena Gelžinytė, Andrea Ardenghi, Saurabh Dixit, Maximilian Obst, Aditha S. Senarath, Niclas S. Mueller, Gonzalo Álvarez‐Pérez, Katja Diaz‐Granados, Ryan A. Kowalski, Richarda Niemann, Felix G. Kaps, Jakob Wetzel, Raghunandan Balasubramanyam Iyer, Piero Mazzolini, Mathias Schubert, J. Michael Klopf, Johannes T. Margraf, Oliver Bierwagen, Martin Wolf, Karsten Reuter, Lukas M. Eng, Susanne C. Kehr, Joshua D. Caldwell, Christian Carbogno, Thomas G. Folland, Markus R. Wagner, Alexander Paarmann

**Affiliations:** ^1^ Fritz‐Haber‐Institut der Max‐Planck‐Gesellschaft Berlin Germany; ^2^ University of Iowa Iowa City USA; ^3^ Paul‐Drude‐Institut für Festkörperelektronik Leibniz‐Institut im Forschungsverbund Berlin e.V. Berlin Germany; ^4^ Vanderbilt University Nashville TN USA; ^5^ Institute of Applied Physics, TUD Dresden University of Technology Dresden Germany; ^6^ Würzburg‐Dresden Cluster of Excellence – EXC 2147 (ct.qmat) Germany; ^7^ Center for Biomolecular Nanotechnologies Italian Institute of Technology Arnesano (Lecce) Italy; ^8^ Department of Mathematical Physical and Computer Sciences University of Parma Parma Italy; ^9^ University of Nebraska Lincoln USA; ^10^ Lund University Lund Sweden; ^11^ Helmholtz‐Zentrum Dresden‐Rossendorf Dresden Germany; ^12^ University of Bayreuth Bayreuth Germany; ^13^ Freie Universität Berlin Berlin Germany

**Keywords:** DFT calculations, FT‐IR, isotopic substitution, near‐field microscopy, shear polaritons

## Abstract

Hyperbolic phonon polaritons ‐ hybridized modes arising from the ultrastrong coupling of infrared light to strongly anisotropic lattice vibrations in uniaxial or biaxial polar crystals ‐ enable to confine light to the nanoscale with low losses and high directionality. In even lower symmetry materials, such as monoclinic β‐Ga_2_O_3_ (bGO), hyperbolic shear polaritons (HShPs) further enhance the directionality. Yet, HShPs are intrinsically supported only within narrow frequency ranges defined by the phonon frequencies of the host material. Here, we report spectral tuning of HShPs in bGO by isotopic substitution. Employing near‐field optical microscopy to image HShPs in ^18^O bGO films homoepitaxially grown on a ^16^O bGO substrate, we demonstrate a spectral redshift of ∼40 cm^−1^ for the ^18^O bGO, compared to ^16^O bGO. The technique allows for direct observation and a model‐free estimation of the spectral shift driven by isotopic substitution without the need for knowledge of the dielectric tensor. Complementary far‐field measurements and ab initio calculations ‐ in good agreement with the near‐field data ‐ confirm the effectiveness of this estimation. This multifaceted study demonstrates a significant isotopic substitution induced spectral tuning of HShPs into a previously inaccessible frequency range, creating new avenues for technological applications of such highly directional polaritons.

## Graphical abstract

Spectral tuning of highly directional hyperbolic shear polaritons is realized via isotopic substitution of ^16^O to ^18^O in monoclinic β‐phase gallium oxide. A red‐shift of almost 40 cm^−1^ is experimentally demonstrated with near‐field imaging, corroborated by the permittivity change extracted from far‐field experiments and density functional theory.

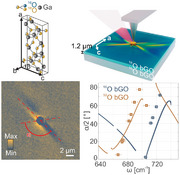

## 1 Introduction

Phonon polaritons, quasiparticles resulting from the hybridization of photons with IR‐active phonon modes, have recently become a milestone in the field of nanophotonics [1]. The reduced optical losses, due to the longer phonon lifetimes compared to their plasmonic counterparts, have rendered them a promising toolbox for applications in nanoscale waveguiding [2, 3], thermal emission [4, 5], heat conduction [6, 7, 8], infrared sensing [9, 10], and subdiffractional imaging [11, 12]. In particular, the discovery of hyperbolicity [13], extensively investigated in materials with hexagonal [11, 14, 15, 16] and orthorhombic [17, 18, 19, 20] crystal structures, has paved the way for new possibilities to achieve deeply sub‐diffractional confinement of light thanks to the large **k**‐vectors made available by the hyperbolic isofrequency contours. In addition, the optical anisotropy arising from the asymmetry in the crystal structure has been shown to be an important factor in enhancing the directionality of polariton propagation [21, 22]. The existence of a plethora of natural highly anisotropic materials, with different intrinsic properties, has provided a diverse scenario for exploring directional phonon polaritons. In this context, the efforts of the scientific community have recently led to the observation of hyperbolic shear polaritons (HShPs) at the interface of monoclinic crystals [23, 24, 25, 26]. HShPs show asymmetric intensity distributions between the two arms of the hyperbola, and dispersive optical axes that are not aligned with the conventional unit cell basis vectors. Furthermore, a number of methods for modifying the directionality of HShPs have been demonstrated, e.g., by employing specifically designed nano‐antennas [25], or by placing a twisted thin slab of an orthorhombic van der Waals (vdWs) material onto the monoclinic substrate [27], inspired by previous studies on twisted α‐MoO_3_ bilayers [28, 29, 30, 31]. Additionally, it has recently been shown that shear polaritons can be engineered by stacking slabs of α‐MoO_3_ with different thicknesses [32].

Despite all these opportunities, a general hurdle of hyperbolic phonon polaritons is represented by the fact that they are supported only in restricted spectral regions referred to as *reststrahlen bands* (RBs) where the dielectric permittivity has opposite signs along the orthogonal crystal axes (or along the major polarizability axes in the case of monoclinic and triclinic crystals [23]). The typical RBs' widths can range from tens to few hundreds of wavenumbers, depending on the oscillator strengths of the phonon modes involved. In the broader context of surface phonon polaritons, which share the same limitation of being defined between the material‐dependent transverse optical (TO) and longitudinal optical (LO) phonon modes, various ways of overcoming the fixed narrow‐band constraint have been explored. To name a few, atomic superlattices consisting of a combination of different polar semiconductors (e.g., GaN, AlN, and SiC) with partially overlapping RBs have been realized [33, 34], extending the available spectral range for SPhPs to over 400 cm^−1^, which represents approximately 160% of the RB width of AlN. Another example consists in the experimental observation of hybridization between HPhPs and surface plasmon polaritons in vdW heterostructures [35, 36, 37, 38, 39], which has caused the dispersion curve to change to the point of exceeding the upper RB limit. Further modulations of the RB have been induced by ion intercalation [40, 41]. Finally, a mass‐dependent shift in the spectral region of interest can be achieved by a more drastic modification of the material and its properties, such as isotopic substitution [42], as so far demonstrated for vdW materials.

Most of the reported work on hyperbolic phonon polaritons in vdW crystals employed thin flakes exfoliated from crystals grown with naturally abundant isotope sources, which typically do not exhibit high degrees of isotopic purity. On the other hand, more recent studies investigated the effects of isotopic enrichment in the same vdW materials. In hexagonal boron nitride (hBN), significantly longer phonon lifetimes and, consequently, longer polariton propagation have been reported for isotopically pure crystals [43]. At the same time, the choice of the boron isotope (^10^B or ^11^B) enabled a shift of the in‐plane TO and LO phonons [43] and, thus, of the polariton band by 30 cm^−1^. Building on this prior work, other studies explored the effect of incorporating the ^15^N isotope in various combinations with high concentrations of boron isotopes on the RB shift, however suggesting minimal changes in the losses [44, 45]. This concept was also extended to broaden the overall polaritonic band by employing isotopically pure heterostructures [46]. For biaxial α‐MoO_3_, the existence of ultralow‐loss phonon polaritons in Mo‐enriched α‐MoO_3_ was experimentally demonstrated, in addition to showing a slight red (blue) shift of polariton bands for the ^100^Mo (^92^Mo) isotope [47, 48].

This study moves away from vdW crystals, focusing instead on the effects of isotopic substitution on the polaritons supported by artificially grown 3D crystals, such as bGO [23, 25] or calcite [21, 22]. The high isotopic purity of commercially grown single crystals of the wide band gap semiconductor bGO is enabling low‐loss propagation of polaritons [23]. In this material, therefore, the substitution of this isotope would allow to shift phonons and polaritons to different frequency ranges while maintaining low losses. Previous work on bGO [49] has shown a significant redshift of approximately 5% for most high‐frequency Raman active phonons [50] when comparing naturally abundant ^16^O and ^18^O. While similar frequency shifts would be expected for the infrared (IR)‐active phonons and the associated polariton bands, this knowledge is still missing. Understanding the effects of isotopic substitution for this material would allow for better control over spectral tuning of polariton bands, thus widening the applicability of bGO in nanophotonic devices.

In this work, we employ far‐infrared near‐field optical microscopy as a tool to demonstrate spectral tuning by approximately 40 cm^−1^ of HShPs on the surface of ^18^O bGO thin films homoepitaxially grown on a ^16^O bGO substrate. The employed experimental method enables real‐space observation of the propagation of strongly anisotropic HShPs in the frequency range of 660–710 cm^−1^. We show that the effect of isotopic substitution can be inferred purely from quantitative analysis of the near‐field images, which provides the dispersion of the optical axes as well as the opening angle of the hyperbola for ^18^O bGO. These two quantities, in fact, enable a model‐free estimation of the relative spectral shift of the polaritons, as well as of the IR‐active phonon modes, caused by isotopic substitution, without requiring knowledge of the dielectric tensor of the (less known) ^18^O isotope. The effectiveness of this estimation is verified by comparing with dielectric model fits to infrared reflectance data and first principles calculations. The ability to evaluate changes in the properties of materials with isotopic purity is particularly important for thin epitaxial films and small‐scale samples – where conventional far‐field infrared characterization is less accurate [51]. Our work introduces and demonstrates isotopic substitution for significant frequency tuning of HShPs in low‐symmetry, polar dielectric 3D crystals, paving the way for integrating shear polaritons efficiently within nanophotonic devices.

## 2 Near‐Field Imaging of Shear Polaritons in Isotopically Substituted bGO

**FIGURE 1:**
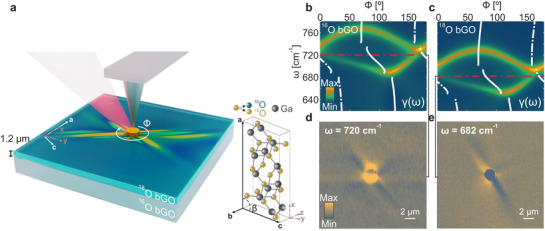
Isotopic substitution‐induced spectral shift of the hyperbolic shear polaritons in β‐Ga_2_O_3_. (a) 3D sketch of the Free‐Electron Laser(FEL)‐coupled s‐SNOM setup, showing the launching of HShPs by a Au disc on the surface of a 1.2 µm thick ^18^O bGO film homoepitaxially grown on a ^16^O bGO substrate. The inset displays a schematic stick‐and‐ball representation of the conventional unit cell of bGO. The lattice constants were determined to be a=12.23Å, b=3.04Å, and c=5.80Å, while the monoclinic angle defined between the *a* and *c* crystallographic axes is $\beta =103.76^{\circ }$ [80]. The monoclinic *a*‐*c* plane coincides with the surface of the sample. (b,c) Azimuthal dispersion for the ^16^O (b) and ^18^O (c) bGO isotopes obtained from transfer matrix simulations at a fixed in‐plane momentum $k_{\mathrm{ip}}/k_{0}=1.1$. The white curves show the axial dispersion, that is the frequency dependence of the optical axis direction, γ(ω), which was calculated using Equation 1 (see Supporting Information, Section S7) with the parameters derived from ab initio theory (see Supporting Information, Table S1 and S2). The red dashed‐dotted lines indicate the frequencies at which the s‐SNOM images shown in panels d,e were acquired. (d,e) Experimental near‐field images of HShPs for the ^16^O (d) and ^18^O (e) bGO isotopes taken at incident frequencies of 720 cm^−1^ and 682 cm^−1^, respectively.

The experimental scheme and major results of spectral tuning of HShPs by isotopic substitution are shown in Figure 1. We employed a commercial near‐field optical microscope (s‐SNOM, *neaSCOPE*) in the self‐homodyne detection scheme coupled to a free‐electron laser (FEL) [52, 53] for near‐field imaging of HShPs. As schematically depicted in Figure 1a, HShPs are launched off a small Au disc of 2 µm diameter acting as an optical nano‐antenna [54, 55, 56, 57] on top of a (010) terminated surface of monoclinic ^18^O bGO. The HShPs are detected interferometrically by measuring the scattered near‐field from a metallic tip scanned over an area of 20 × 20 µm^2^ (see Methods for more details on the experiment). The inset of Figure 1a shows a schematic stick‐and‐ball representation of the conventional unit cell of bGO. The monoclinic a–c plane (010) is parallel to the surface in our experiment. Since ^18^O bGO bulk single crystals are not readily available, we employ molecular beam homoepitaxy [50] to grow a strain‐free, lattice‐matched 1.2 µm thick ^18^O bGO film onto a commercial ^16^O bGO (010) substrate. The choice of this specific thickness is explained in SI section S4, where s‐SNOM images of HShPs on ^18^O bGO films of different thicknesses are shown for comparison. By employing nano‐antennas that only excite high momenta of the HShPs, the polaritonic near‐fields cannot reach the ^16^O bGO substrate, and thus, the experiment solely probes the hyperbolic surface modes of ^18^O bGO.

In the following, we illustrate the spectral tuning of HShPs driven by isotopic substitution, as anticipated from the observed 5% shift in the Raman active modes [50]. To this end, we plot the simulated azimuthal dispersion maps [23, 58] for HShPs in Figure 1b,c for ^16^O (b) and ^18^O (c) bGO for a fixed in‐plane polariton momentum $k_{\mathrm{ip}}/k_{0}=1.1$ (thus assuming low confinement, as $k_{0}$ is the momentum of light in vacuum), see Methods for details. Note that these maps do not consider the epitaxial heterostructure, but were calculated for semi‐infinite crystals. Both polariton dispersion plots are notably asymmetric due to the shear effect [23], arising from an imbalanced intensity distribution across the two arms of the hyperbolic isofrequency contours in momentum space. A close examination reveals that the two plots are nearly identical with the exception of a significant frequency shift of approximately 40 cm^−1^ between both maps. The spectral shift is further highlighted by the analytical plots of the axial dispersion angle γ(ω), that represents the frequency dependence of the optical axis orientation (calculated using Equation 1 below, for details see Section S7, Supporting Information), indicated by white curves overlaid with the simulations. To directly probe the resonances (defined in the regions of high intensity in the simulated dispersion maps) would require a prism‐coupling experiment [23]. However, due to its intrinsic limitation, this experimental technique can only access low momenta, hence the low confinement assumed in the simulations. In the presence of a few µm thick epitaxial layer, as in our experiment, the evanescent fields of surface‐bound HShPs would penetrate into the ^16^O substrate, dramatically complicating any analysis. Instead, we employed near‐field imaging of the strongly anisotropic HShP propagation launched by nano‐antennas, as illustrated in Figure 1d,e. This technique allows for access to higher momenta. Panels d,e show qualitatively nearly identical near‐field patterns with hyperbolic rays along very similar directions and comparable length for both isotopes. Yet, these very similar patterns emerge with a frequency shift of 38 cm^−1^ between them in agreement with the dispersion plots in Figure 1b,c.

**FIGURE 2:**
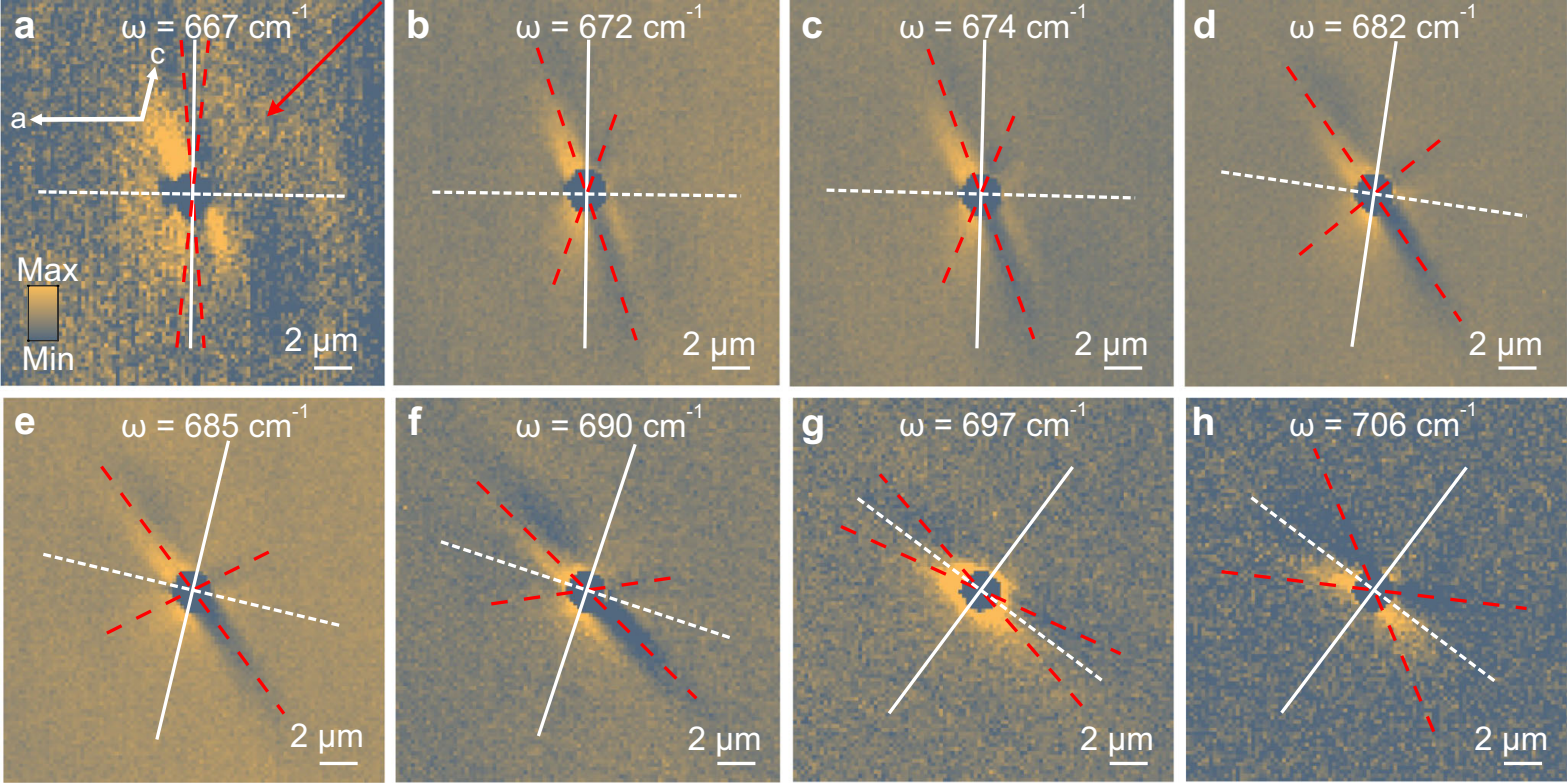
Near‐field propagation of low‐symmetry hyperbolic polaritons in isotopically substituted ^18^O bGO film on ^16^O bGO substrate. (a–h) Near‐field microscopic images of HShPs in a 1.2 µm thick isotopically substituted bGO film homoepitaxially grown on a ^16^O bGO substrate. The excitation of the modes occurs though a Au disk with a 2 µm diameter. The panels show images recorded at different excitation frequencies. For all images, we show the optical amplitude demodulated at the second harmonic of the tip tapping frequency (O2A). The signal was acquired in the self‐homodyne detection scheme. The red dashed lines indicate the asymptotes of the polariton wave fronts, see Supporting Information Section S1 for details on their derivation. The white lines represent the two major polarizability axes of the material system. Panel (a) contains information on the illumination direction (red arrow) and on the crystallographic axes orientation (white arrows), see Supporting Information Section S9.

In order to quantitatively analyze the spectral tuning of HShPs due to the isotopic substitution, we acquired near‐field images of HShPs bound to the air/^18^O bGO interface at several different frequencies as summarized in Figure 2. For these measurements, we keep the azimuthal orientation of the sample fixed with regard to the incident beam as illustrated in Figure 2a, resulting in a propagation pattern with very large asymmetry arising from intrinsic shear‐induced, as well as illumination‐induced, symmetry breaking [25, 59]. Furthermore, the 1.2 µm thick ^18^O bGO epilayer acts *de facto* as a semi‐infinite bulk crystal by supporting surface modes similar to previous work using ^16^O bGO substrates [25]. This is achieved here by employing 2 µm diameter Au disks as nano‐antennas that efficiently excite HShPs with high momenta (∼10 $k_{0}$), resulting in evanescent waves with a skin depth of about 0.5 µm into the film, see Section S10 (Supporting Information) for details. In contrast, larger antennas excite lower momentum near‐fields [25] that penetrate deep enough into the film to reach the substrate, resulting in peculiar patterns that are more difficult to interpret, as discussed in detail in Section S3 (Supporting Information). Furthermore, exciting only large‐momentum components of the HShPs also results in a ray‐like propagation pattern with the direction of the rays coinciding with asymptotes of the in‐plane hyperbolic wave fronts [25, 59]. These experimental design choices significantly reduce the complexity of the s‐SNOM images, and enable a thorough quantitative analysis of the real‐space polariton propagation – a nontrivial achievement in the context of low‐symmetry polaritons [17, 21, 25, 59].

The angle between the rays in the real‐space polariton propagation is directly linked to the opening angle α(ω) of the respective in‐plane hyperbolic isofrequency surface in momentum space [17, 60]. This is in turn determined by the in‐plane anisotropy in the permittivity [60] (see Equation 2 later in the manuscript). We extracted the direction of both polariton rays from each image, as displayed by the dashed red lines in Figure 2a–h, see Sections S1.1–S1.3 (Supporting Information) for details on the fitting procedure. With both rays extracted, we can also experimentally determine the direction of the optical axes, located exactly in the middle between both rays, see solid and dashed white lines in Figure 2a–h. Since the rays correspond to the asymptotes of the in‐plane hyperbolic wave fronts in momentum space, we can infer that the optical axes coincide with the hyperbola symmetry axes [23]. Notably, both parameters – optical axes and polariton rays directions – are determined from the near‐field data without assuming any physical model, and allow for a quantification of the frequency shift from isotopic substitution. Furthermore, these quantities can be linked directly to the in‐plane permittivity of ^18^O bGO. However, so far the permittivity tensor of the ^18^O bGO isotope has not been reported in the literature.

## 3 Anisotropic In‐Plane Permittivity of ^18^O bGO

**FIGURE 3:**
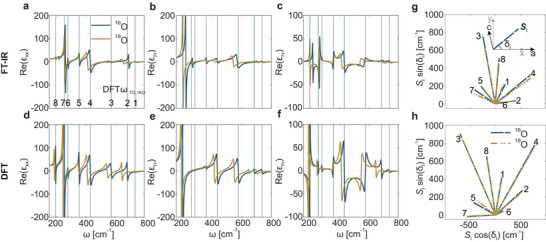
Experimental and theoretical permittivity data for the ^16^O and ^18^O bGO. (a–f) Real parts of ɛ_xx_, ɛ_yy_, and ɛ_xy_ for ^16^O and ^18^O bGO, blue and orange lines, respectively, as extracted from azimuth‐dependent polarized reflection measurements (a–c) and ab initio calculations (d–f). The solid vertical lines indicate the positions of the theoretically derived TO phonon frequencies for the ^16^O bGO isotope. The numbers refer to the IR active modes with *B*_u_ symmetry and are consistent with the notation used in Table 1. (g) Experimentally (FT‐IR) and (h) theoretically (DFT) derived oscillator orientation vectors $S_{i}$ of the *B*_u_ modes in the monoclinic a‐c plane.

To corroborate the near‐field extraction of the frequency shift induced by the isotopic substitution, we performed polarized far‐field reflectance measurements as well as density‐functional theory (DFT) calculations. By means of these two methods, we could experimentally and theoretically determine the in‐plane permittivity of ^18^O bGO, and compare these results to respective data for ^16^O bGO, see Figure 3.

The polarized reflectance measurement was conducted with a commercial Fourier‐transform infrared (FT‐IR) spectrometer (*Bruker Vertex 80V*) to obtain reflectance spectra at multiple azimuthal angles (see Methods for more details [51]). The measurements were performed using the same sample employed for the near‐field microscopy [1.2 µm ^18^O bGO epitaxially grown on a ^16^O (010) bGO substrate], as well as a comparable ^16^O (010) bGO substrate. From fits of the reflectivity spectra, we obtain the in‐plane permittivity components of ^16^O and ^18^O bGO, shown as orange and blue curves in the plots in Figure 3a–c, respectively.

It is important to note that, because of the monoclinic crystal structure of bGO, the atomic displacements of the $B_{u}$ phonon modes are not aligned with the crystal axes. As a consequence, the oscillator orientation vector $S_{i}$ associated with a given phonon mode forms a non‐trivial (neither parallel nor perpendicular) angle $\delta_{i}$ with the crystallographic axis a, as shown in the inset of Figure 3g. Note that, within the reference system under consideration, the *a*‐axis is oriented in a parallel alignment with the *x*‐axis, while the *c*‐axis forms an angle of 13.76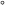 with the *y*‐axis.

The experimentally derived oscillator orientation vectors are plotted in Figure 3g; their magnitude is quantified by the oscillator strength $S_{i}$. The TO phonon frequencies $\omega_{\mathrm{TO},i}$, the oscillator strengths $S_{i}$, the angles $\delta_{i}$, and the damping constants $\gamma_{i}$ were derived from a global fit using the commercial software *WVASE* for all eight in‐plane IR‐active modes with *B*_u_ symmetry. The full set of fitting parameters along with the corresponding fitting errors is reported in the Supporting Information, Table S1 (see also Table S2 for the electronic contributions), for the ^18^O film and the ^16^O substrate. For more information on the experimental fitting procedure, see Section S6 (Supporting Information).

The theoretical results, obtained from the harmonic, anharmonic, and dielectric properties computed via DFT as explained in the Methods section, are plotted alongside the experimental data in Figure 3d–h. The calculated $S_{i}$ of the *B*_u_ modes for both isotopes are plotted in Figure 3h. The full DFT data are reported in Supporting Information, Table S1 and S2, alongside the experimental values for a direct comparison.

Overall, the theoretically predicted TO phonon frequencies demonstrate a high degree of correlation with the FT‐IR measurements, with maximum deviations of less than 10 cm^−1^, comparable to previous work on ^16^O bGO [61] (see vertical lines in Figure 3a‐f). Conversely, DFT calculations generally predict larger oscillator strengths in comparison to the experimental parameters. In addition, the mode orientations, $\delta_{\text{i,th}}$, predicted by ab initio calculations appear rotated around 20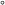 counterclockwise relative to those determined from the FT‐IR reflectance maps, $\delta_{\text{i,exp}}$, as illustrated by the **S**_i_‐vector orientation in Figure 3g,h. Despite these discrepancies, theory and experiment reveal a comparable trend insofar that the major effect of the isotopic substitution is observed in the phonon frequencies, while the oscillator orientation vectors are largely unaffected. This implies that the HShPs, which arise from the permittivity of the material, will also predominantly experience a spectral shift while propagation characteristics are maintained during isotopic substitution. To quantify the spectral shift of the phonon frequencies, we evaluated the relative spectral shift for the *B*_u_ modes, $\Delta \omega_{rel.}$, as provided in Table 1. Consistent between experiment and theory, as well as compared to Raman data [50], we find a spectral shift of approximately 5% for the high frequency modes (#1−#4), and a smaller shift of only 1% in modes #5 and #6, with somewhat poor agreement for the low‐frequency modes #7 and #8. This discrepancy can be attributed to the cutoff in the FT‐IR reflectance measurements at 200 cm^−1^, which affects the fitting results for the damping constants and oscillator strength values for these modes (see Supporting Information, Section S6). Nevertheless, the good overall agreement in relative spectral shifts between the far‐field and DFT results further validates the impact of isotopic substitution on the dielectric tensor of monoclinic crystals.

**TABLE 1:** Oxygen isotope effect on in‐plane transverse optic phonons in bGO. The relative frequency shifts of TO phonons polarized in the monoclinic plane ($\Delta \omega_{rel.}$) were obtained from the experimentally and theoretically derived TO phonon frequencies, as $\frac{\omega_{\text{TO,16}}-\omega_{\text{TO,18}}}{\omega_{\text{TO,16}}}$. The B_u_ modes frequencies for the ^16^O bGO isotope are reported alongside the relative spectral shifts, for a direct comparison between FT‐IR measurements and DFT calculations. A full set of parameters is provided in the Supporting Information, Table S1 and S2.

	B_u_ mode	#1	#2	#3	#4	#5	#6	#7	#8
FT‐IR	$\Delta \omega_{rel.}$ [%]	5.2	5.6	5.1	4.4	0.9	0.8	2.1	9.2
	$\omega_{\text{TO,16}}$ [cm^−1^]	742.(5)	694.(0)	567.(2)	430.(0)	356.(2)	278.6(4)	260.(5)	214.(8)
DFT	$\Delta \omega_{rel.}$ [%]	5.5	5.4	4.9	4.9	1.3	1.1	3.4	4.9
	$\omega_{\text{TO,16}}$ [cm^−1^]	737.28	684.17	574.22	430.40	354.73	277.30	252.68	193.74

Importantly, we expect the highest frequency modes #1 and #2 to be dictating the permittivity responsible for formation of the HShPs [62] observed in Figure 2, suggesting that we can predict a spectral tuning of these modes of approximately 5.5%, corresponding to Δω∼ 40 cm^−1^ at ω= 685 cm^−1^ in the middle of the hyperbolic polariton band.

## 4 Spectral Tuning of Shear Polaritons

**FIGURE 4:**
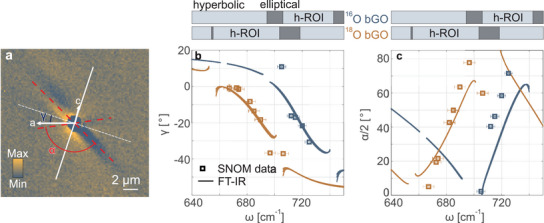
Experimental verification of the spectral tuning of the optical axis dispersion γ and the hyperbola opening angle α by isotopic substitution. (a) Illustrative sketch of the angles γ and α. While γ is defined between the crystallographic a‐axis and the major polarizability axis (white dashed line), the opening angle α is defined between the two polariton rays (red dashed lines). As an illustrative example, we have chosen a s‐SNOM image for an incident frequency of 690 cm^−1^. (b,c) Optical axis dispersion angle γ (b) and opening angle α (c) for the ^16^O (blue curve) and ^18^O (orange curve) isotopes of bGO. Orange squares indicate the near‐field results relative to the epitaxial heterostructure reported with the corresponding error bars (see Supporting Information Section S8), extracted from the s‐SNOM images in Figure 2, see Supporting Information Sections S1‐S3 for a full analysis. The blue squares were extracted from the s‐SNOM images relative to ^16^O bGO reported in the Supporting Information, Figure S5.1, following the same analysis procedure. The solid curves have been derived analytically [23, 60] from the experimental permittivity data (shown in Figure 3), and are reported with their broadening obtained from the experimental uncertainty on the polarized FT‐IR measurements (see Supporting Information Section S8). The gray‐shaded bars above panels b and c show the spectral ranges where different types of phonon polaritons are supported, see Supporting Information Figure S7.4. h‐ROI indicates the hyperbolic range of interest, where HShPs are defined.

To further quantify the spectral tuning of HShPs in bGO via isotopic substitution, we compare the angles γ and α extracted from the near‐field images in Figure 2 with those calculated analytically using the FT‐IR‐extracted permittivity (Figure 3). The results of this comparison are presented in Figure 4.

While γ(ω) is defined between the crystallographic a‐axis and the major polarizability axis (white dashed line), the opening angle α(ω) spans between the two polariton rays (red dashed lines). Figure 4a contains an illustrative sketch of the two angles. In Figure 4b, we show values for γ for ^18^O bGO extracted from the data shown in Figure 2, as well as for ^16^O bGO (see Supporting Information, Section S5 for the respective near‐field images), as orange and blue square symbols, respectively. Indeed, these near‐field results show excellent agreement with the corresponding curves for γ extracted analytically from the FT‐IR obtained permittivity data (Figure 3a–c) displayed as lines with their broadening indicating the error margins. In contrast to previous studies [23, 25], here the analytical curves for γ are calculated using the arctan2 function, which allows for 2π periodicity of the optical axis rotation across the entire spectral range:

(1) $$\gamma \left(\omega \right)=\text{arctan2}\left(m_{x},m_{y}\right)$$

The arguments $m_{x}$ and $m_{y}$ are the x‐ and y‐coordinates of the major polarizability axis, m, i.e., the principal eigenvector of $ℜ(\varepsilon_{\text{xy}})$.

Similarly, we plot the near‐field data (square symbols) and the analytical values obtained using the FT‐IR permittivity (continuous curves), for the half‐opening angle α/2 in Figure 4c. To compute the angle α we make use of a generalization of the expression derived for in‐plane hyperbolic, biaxial crystals [60]:

(2) $$\alpha \left(\omega \right)=2\;\text{arctan}\left(\sqrt{-\frac{R\left(\varepsilon_{\text{nn}}\left(\omega \right)\right)}{R\left(\varepsilon_{\text{mm}}\left(\omega \right)\right)}}\right)$$

where $\varepsilon_{\text{mm}}\left(\omega \right)$ and $\varepsilon_{\text{nn}}\left(\omega \right)$ are the in‐plane permittivity tensor components in the frequency‐dependent coordinate system defined by the major polarizability axes [23]. Also for α, the near‐field observation shows excellent agreement with the analytical prediction (Figure 4c). See Supporting Information, Section S7 for details on the analytical derivation of γ and α.

As predicted by ^16^O and ^18^O bGO permittivities from FT‐IR measurements, we observe HShPs in the spectral range spannig approximately 40 cm^−1^, bounded by elliptical polaritonic ranges. The hyperbolic and elliptical bands are visualized in Figure 4b,c (top), where they are depicted as light‐blue and grey‐shaded horizontal bars, respectively, thereby delineating the hyperbolic range of interest (h‐ROI) where we experimentally observed HShPs. The hyperbolic and elliptical bands were identified based on the rotated (in‐plane) ɛ‐tensor components [23], i.e., ɛ_mm_ and ɛ*_nn_*, see Supporting Information Figure  S7.4 for details. Strikingly, the extracted parameters γ and α fully characterize the propagation characteristics of the polaritons and, thus, the near‐field data shown in Figure 4 unambiguously prove the spectral tuning of HShPs, and consequently of the TO phonon frequencies, by isotopic substitution. This finding is based solely on direct observations of polariton propagation in real space. Although it is useful for proving the technique's effectiveness in quantifying the correct spectral shift, knowledge of the relevant isotope's dielectric tensor is not strictly necessary.

## 5 Conclusion

In summary, we used near‐field optical microscopy to study the real‐space propagation of surface‐bound HShPs in a 1.2 µm thick ^18^O bGO film homoepitaxially grown on a ^16^O bGO substrate. We compared these near‐field data with far‐field measurements and ab initio calculations, showing excellent agreement. The combined and comprehensive study demonstrates a spectral tuning of approximately 40 cm^−1^ for shear polaritons in bGO using isotopic substitution, while all other polariton characteristics remain largely unchanged. Furthermore, our findings show that extracting information on polariton propagation directly from near‐field data allows for precise estimation of the spectral shift induced by isotopic substitution without requiring a full measurement of the dielectric tensor. This type of determination is particularly relevant for the analysis of vdW crystals or thin epitaxial layers, where traditional far‐field IR spectroscopy has inherent limitations. In general, our work demonstrates an exceptional spectral tunability of highly directional HShPs enabled by isotopic substitution, thereby unveiling novel avenues for the exploration of these phenomena at hitherto inaccessible frequencies, and suggests the possibility of its quantification solely with near‐field imaging.

## 6 Methods

### 6.1 Sample Preparation

The ^18^O bGO sample was grown in a plasma‐assisted molecular beam epitaxy (PA‐MBE) chamber equipped with an RF‐plasma source (SPECS PCS). The deposition was made on a 5x5 mm^2^ β‐Ga_2_O_3_ (010) Fe‐doped substrate purchased from Novel Crystal Technology. Prior to the growth, the substrate was sequentially solvent cleaned using acetone and isopropyl alcohol (IPA) for 5 minutes with sonication, followed by 30 min of $O_{2}$ plasma treatment (300 W, 1 standard cubic centimeter per minute (SCCM)) in the growth chamber at a substrate temperature of 800 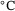. The growth was performed at a substrate temperature of 700 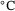 by simultaneously supplying elemental gallium from a double filament effusion cell, with a corresponding beam equivalent pressure (BEP, measured by a nude ion gauge at the substrate position) of BEP_Ga_ = 3.4 × 10^−7^ mbar, and 1 standard cubic centimeter per minute (1 SCCM) of isotopically enriched oxygen (^18^O_3_) with a purity of 97.39% at 250 W of RF plasma power. The layer thickness was measured using profilometry. We note that also a thinner and a thicker film of ^18^O bGO were investigated using s‐SNOM but yielded inferior results, see Figure S4.1.

The Au antennas were fabricated on the surface of the ^18^O bGO sample by a standard electron beam lithography process. A two‐layer PMMA resist stack was prepared by sequentially spin‐coating AR‐P 662.04 and AR‐P 679.04 over an adhesion promoter (AR 300‐80) followed by soft bake. A conductive Electra coating was applied to prevent charging during exposure. After exposure, the conductive layer was rinsed off, and the resist was developed with AR 600‐56 developer, followed by IPA. Metal deposition of Cr/Au (5 nm/50 nm) was followed by lift‐off in acetone in an ultrasonic bath. We note that small circular shape imperfections of the antennae are not expected to affect the propagation patterns due to their deeply sub‐diffractional size [63].

### 6.2 FEL s‐SNOM Measurements

The near‐field images illustrated in Figure 2 were acquired by means of a commercial s‐SNOM system from *neaSPEC* (now part of *attocube systems*) coupled to the free‐electron laser FELBE at Helmholtz Zentrum Dresden‐Rossendorf (HZDR). FELBE was operated at a repetition rate of ∼13 MHz, with a spectral bandwidth of about 0.5%. For the complete dataset, the incident light was p‐polarized. The measurements were performed using a mercury‐cadmium‐telluride (MCT) detector in a self‐homodyne detection scheme, due to the comparatively low signal‐to‐noise ratio of the light source that does not allow reliable operation using the pseudo‐heterodyne method. In this configuration, the detected signal contains a mixture of near‐field and background contributions and the optical amplitude and phase are mixed and cannot be separated [6]. Still, an analysis of the features of the HShPs separate to the background contributions was possible due to their high‐momentum nature compared to the low momenta dominating the background. More details on the particular setup are available in previous publications [25, 64].

### 6.3 Transfer Matrix

The azimuthal dispersion maps in Figure 1b,c were derived from transfer matrix (TM) simulations [58], a formalism that allows for numerical evaluation of the reflection and transmission coefficients in multilayered media. The quantity plotted is the imaginary part of the Fresnel reflection coefficient for p‐polarized light, $ℑ(R_{\mathrm{pp}})$, normalized to its maximum. As shown in a previous work [65], the high intensity regions emerging from these plots are suitable for potential excitation of surface‐bound polaritonic modes, since the maxima of $ℑ(R_{\mathrm{pp}})$ coincide with the material's resonant modes.

### 6.4 FT‐IR Measurements

The Fourier Transform Infrared (FT‐IR) spectroscopy measurements were performed for the ^16^O bGO substrate and the isotopically enriched ^18^O bGO film sample using a commercial FT‐IR spectrometer (Bruker Vertex 80V) with a modified sample holder (A513/QA). Polarized reflectance spectra for both samples were measured at varying azimuthal orientations, ranging from 0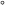 to 180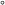 with 5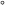 increments. The incidence angle was fixed for each azimuthal measurement: 50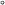 for ^16^O bGO, and 15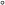 for the ^18^O bGO epitaxial layer. The optical measurement layout consists of a deuterated L‐alanine doped triglycine sulphate (DLaTGS) IR detector, a KRS5 polarizer, and a Si beam splitter.

### 6.5 Dielectric Tensor Formula for Monoclinic Crystals

The in‐plane dielectric tensor components for monoclinic crystals are derived from the Lorentz oscillator model through the following equation [61]:

(3) $$\varepsilon \left(\omega \right)=\varepsilon_{\infty }+\sum_{i}^{N_{\mathrm{phonon}}}\frac{S_{i}⊗S_{i}^{*}}{\omega_{\mathrm{TO},i}^{2}-\omega^{2}-i\omega \gamma_{i}}$$

where $\varepsilon_{\infty }$ denotes the electronic contribution to the dielectric permittivity, $S_{i}$ the oscillator orientation vector, $\omega_{\mathrm{TO},i}$ the transverse optical phonon frequency, and $\gamma_{i}$ the damping constant. The summation is performed over the eight IR‐active phonon modes that are defined in the monoclinic plane. The experimentally derived parameters for the dielectric tensor components plotted in Figure 3, using Equation 3, are reported in Supporting Information, Table S1 and S2. The angle between the x‐axis and the vector $S_{i}$ associated with the phonon mode i is computed as $\delta_{i}=\arctan\left(S_{\text{i,y}}/S_{\text{i,x}}\right)$. It should be noted that the oscillator strengths, $S_{i}$, are expressed in units of cm^−1^.

### 6.6 DFT calculations

To assess the properties of phonon polaritons in bGO from first principles, all parameters entering the Lorentz oscillator model defined by Equation (3) are calculated via DFT and, as appropriate, density‐functional perturbation theory (DFPT) [66] using the FHI‐aims code [67, 68]. All presented results were obtained using a 11×6×6 momentum **k**‐grid in the first Brillouin zone associated with the primitive unit cell (a=3.02Å, b=5.76Å, c=6.26Å, $\alpha =103.32^{\circ }$, $\beta =103.96^{\circ }$, $\gamma =90^{\circ }$), “tight” defaults for the basis set and the numerical settings [67, 69], and the local density approximation in the parametrization proposed by Perdew and Zunger [70] for modeling exchange and correlation, in line with earlier DFT studies of bGO [61].

More specifically, bGO was first relaxed under symmetry constraints [71] until the largest (generalized) force component associated with the atomic and lattice degrees of freedom fell below 0.001 eV/$\overset{˚}{A}$. For this optimized structure, the electronic contribution to the dielectric permittivity ($\varepsilon_{\infty }$) was calculated with DFPT [72], while the harmonic phonon properties (including the angular frequencies $\omega_{\mathrm{TO},i}$) and the first‐order anharmonic corrections defining the damping constants $\gamma_{i}$ were computed using finite displacements in a 4×2×2 supercell. The phonon eigenvectors ${\hat{e}}_{i\kappa }$ of the dynamical matrix at Γ were used to compute the oscillator orientation vectors defined as [66]

(4) $$S_{i}^{\mathrm{DFT}}=\frac{1}{c\sqrt{\varepsilon_{0}V}}\sum_{\kappa }^{N_{\mathrm{atoms}}}\frac{1}{\sqrt{M_{\kappa }}}Z_{\kappa }{\hat{e}}_{i\kappa },$$

where c denotes the speed of light, $\varepsilon_{0}$ the free‐space permittivity, V the volume of the unit cell, $M_{\kappa }$ the mass of the atom with index κ, and $Z_{\kappa }$ its Born effective charge (tensor).

We chose a computational approach (LDA exchange‐correlation functional, finite‐difference phonons in the 0 K limit, no LO‐TO corrections [66]) that is consistent with earlier works on phonon‐polaritons in bGO [61], since isotopic spectral shifts are rather insensitive to these details. Still, an even better quantitative agreement with experiment for absolute peak positions can be achieved by improving on these approximations, e.g., by employing advanced electronic‐structure theory, by accounting for anharmonic shifts, and by explicitly including phonon‐photon coupling [73].

The Born effective charges were also computed via finite differences, i.e., by monitoring the change in polarization [74] upon the displacement of atom κ [75]. By using these definitions, both $S_{i}$ and ε(ω) are directly comparable between theory and experiment and the effects of the isotopic substitution can be straightforwardly obtained by changing the atomic masses $M_{\kappa }$ in Equation (4) and in the (an)harmonic phonon calculations. The wfl workflow management package [76] and the FHI‐vibes [77] interface to the implementations available in Phonopy [78] and Phono3py [79] were used for geometry optimization and to compute the phonon properties.

## Conflicts of Interest

The authors declare no conflict of interest.

## Supporting information


**Supporting File**: adma71816‐sup‐0001‐SuppMat.pdf

## Data Availability

The data that support the findings of this study are openly available in Zenodo at https://doi.org/10.5281/zenodo.17978311.
