# Supplementary material for: Spectral Tuning of Hyperbolic Shear Polaritons in Monoclinic Gallium Oxide via Isotopic Substitution
Source: Adv Mater. 2026 Jan 10;38(11):e14561. doi: 10.1002/adma.202514561 (PMC12921354; doi:10.1002/adma.202514561)
Supplement: Supplementary file 1 — Supporting File: adma71816‐sup‐0001‐SuppMat.pdf [file ADMA-38-e14561-s001.pdf]

Spectral tuning of hyperbolic shear polaritons in monoclinic gallium oxide via isotopic substitution. Supplementary Information

Giulia Carini[1], Mohit Pradhan[2], Elena Gelžinytė[1], Andrea Ardenghi[3], Saurabh Dixit[4], Maximilian Obst[5], Aditha S. Senarath[4], Niclas S. Mueller[1], Gonzalo Alvarez-Perez[6], Katja Diaz-Granados[4], Ryan A. Kowalski[4], Richarda Niemann[1], Felix G. Kaps[5], Jakob Wetzel[5], Raghunandan Balasubramanyam Iyer[2], Piero Mazzolini[3], Mathias Schubert[7], J. Michael Klopf[8], Johannes Margraf[9], Oliver Bierwagen[3], Martin Wolf[1], Karsten Reuter[1], Lukas Eng[5], Susanne Kehr[5], Joshua D. Caldwell[4], Christian Carbogno[1], Thomas G. Folland[2], Markus R. Wagner[3], and Alexander Paarmann[1]

[1]Fritz Haber Institute of the Max Planck Society, Berlin, Germany
[2]University of Iowa, Iowa City, IA, USA
[3]Paul Drude Institute for Solid State Electronics, Berlin, Germany
[4]Vanderbilt University, Nashville, TN, USA
[5]TUD Dresden University of Technology, Dresden, Germany
[6]Italian Institute of Technology, Lecce, Italy
[7]University of Nebraska, Lincoln, NE, USA
[8]Helmholtz-Zentrum Dresden-Rossendorf, Dresden, Germany
[9]University of Bayreuth, Bayreuth, Germany

October 24, 2025

S1 s-SNOM image analysis

In this section, we provide a detailed description of the data treatment performed to derive the frequency-dependent rotation angle γ and the opening angle α from the s-SNOM images shown in the main text (Fig. 2). The experiment was carried out using the self-homodyne detection scheme, i.e. the reference arm of the interferometer in the s-SNOM setup was blocked during the scans. This detection scheme provides only an intensity measurement, while the near-field phase information cannot be decoupled from the background phase [4]. The plots in Fig. 2 display the amplitude of the optical signal demodulated at the 2nd harmonic of the cantilever oscillation frequency. We found the demodulation at the second harmonic to be sufficient to extract the near-field contribution and all optical images analyzed in this work were demodulated at this harmonic. In order to obtain a quantitative estimate of the polariton orientation from these images, several levels of analysis were performed that are exemplified in the following for an incident wavelength of $682\,\text{cm}^{-1}$.

S1.1 Identification of Au discs position

First, the position of the Au discs was determined to identify the region where the polariton is launched. This was achieved by analyzing the atomic force microscopy (AFM) images provided by the *neaSCOPE* device (from

neaSPEC, now part of *attocube systems*) simultaneously with the near-field scans; this allows fair referencing. To find the coordinates of the centre of the disks (x_0, y_0), we identified the rising and falling edges of the peaks in the topography using Matlab's 'findpeaks' function and calculated the midpoint between them. All pixels in the AFM and optical images were then shifted accordingly to re-centre the disc. In Figs. S1.1 a-b we have superimposed the corrected AFM (a) and near-field optical images (b) with a circle (red solid line) of radius 1 µm centred at the origin.

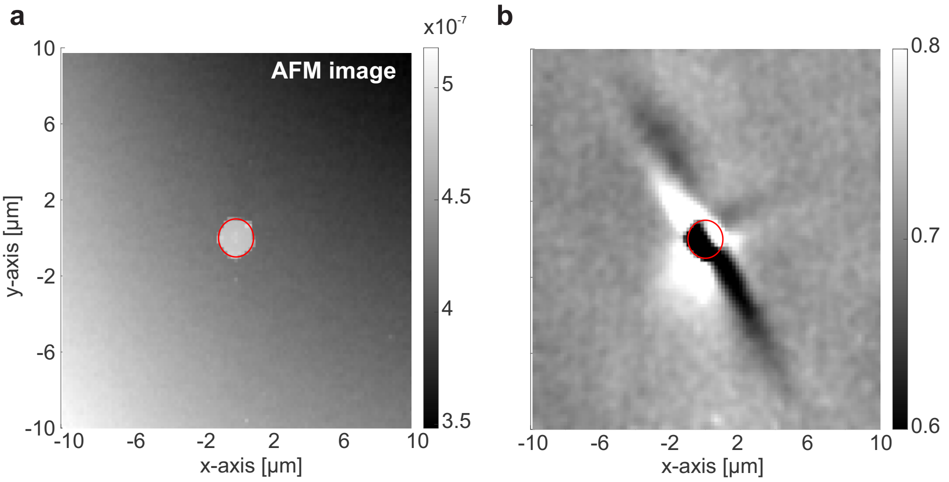

Figure S1.1: **Identification of Au disc position**. (a,b) AFM (a) and s-SNOM (b) images after being re-centred. The pixels were shifted by the disc centre coordinates (x_0, y_0). Both images are overlaid with a circle of radius 1 µm centred on the origin (red solid line).

S1.2 Analysis of the polariton propagation direction

Fig. S1.2 describes the subsequent steps of analysis. The raw near-field scan for an incident frequency of 682 cm^{-1} is shown in panel a, while in panel b a gauss filter was applied to the image to smooth the data, reducing the noise and simplifying the following data treatment. Note that the polariton in Figs. S1.2 a-b is launched from a Au disc of 2 µm diameter. As shown in a previous paper [5], the size of the antenna acts as a momentum filter and plays an important role in determining the anisotropy of the resulting polariton wave (see Section S3). Small discs, like the one we chose for our measurements, allow the selection of higher momenta. Due to the asymmetric intensity distribution along the two arms of the hyperbola in momentum space, this leads to a ray-like polariton propagation in real space [2]. The absence of fringes simplifies the determination of the polariton rays direction, which we derive from the dips in the near-field optical intensity signal (S_2).

Since the polariton waves are emitted from the edges of the antenna, the rays we observe are expected to be orthogonal to a circle centred on the origin. Therefore, the easiest method to extract the polariton propagation direction is to treat the rays as vertical lines in the near-field optical image, once converted from Cartesian to polar coordinates, as shown in Fig. S1.2 c. The conversion was performed by first transforming the coordinates (x, y) of each pixel of the image in panel b into the corresponding polar coordinates (ρ, θ) and then interpolating (with Matlab's function 'scatteredInterpolant') for new axes ρ_{eq} and θ_{eq} with equidistant points.

This coordinate transformation allowed for a more straightforward extraction of the dip positions in the near-field intensity signal. The image was divided into three different angular regions (corresponding to a short and two long rays) and horizontal line cuts were applied at each ρ_{eq} value. By applying Matlab's 'findpeaks' function to $\mathrm{Max}(S_2) - S_2$ (where S_2 is the intensity of the near-field optical signal demodulated at the second harmonic), we were

able to extract the near-field intensity dips along with their full width at half maximum (FWHM). To ensure that the false dips were discarded, we set appropriate thresholds for minimum peak prominence and maximum peak width, and then selected the dip with the largest peak prominence among those extracted by the function. Thus, for each angular region, we obtained an array of corresponding dip positions and FWHMs. If the criteria mentioned above were not met, NaNs were written as array elements instead. This led us to define several segments between the NaNs, among which the longest one was selected for each polariton ray. The dip positions along with the corresponding FWHM are shown in Fig. S1.2 c, overlaid with the result of the interpolation discussed above.

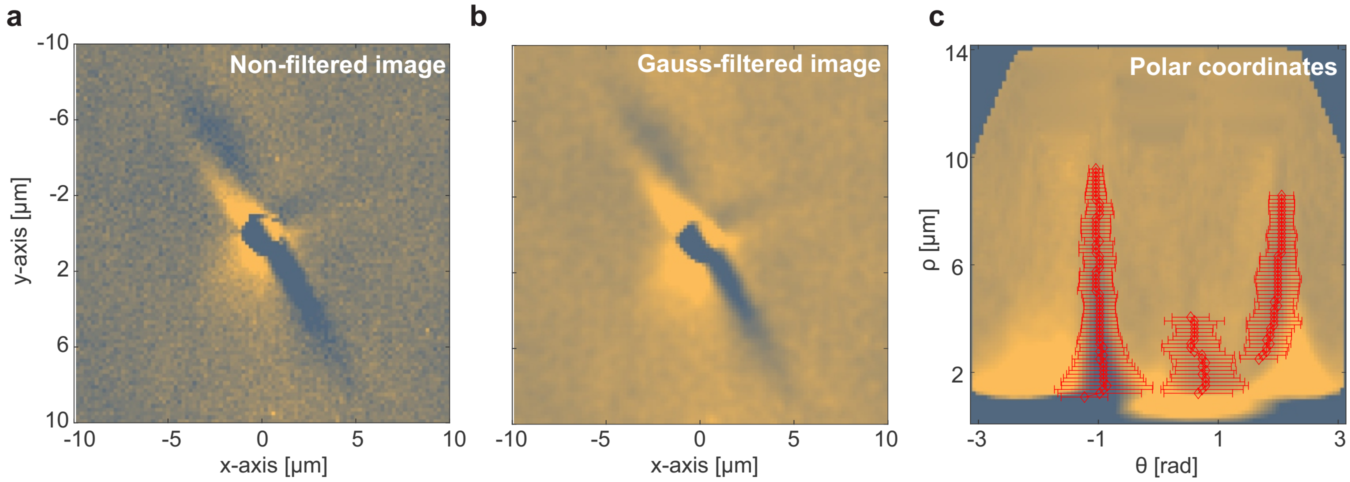

Figure S1.2: **Analysis of the polariton propagation direction**. (a-b) Raw (a) and gauss-filtered (b) near-field optical images demodulated at the second harmonic for an incident wavelength of 682 cm^{-1}. The pixels had been shifted by the disc centre coordinates (x_0, y_0) (see Fig. S1.1). (c) Optical image converted to polar coordinates, overlaid with the dip positions of the longest segments for each 'angular' region. The error bars (red solid lines) correspond to the FWHM of the extracted dips.

S1.3 Analysis extended to various incident FEL frequencies

The procedure illustrated in the previous sections for one frequency of the incident FEL beam is now applied to various frequencies. Fig. S1.3 shows the s-SNOM images after conversion into polar coordinates, interpolation for the axes ρ_{eq} and θ_{eq}, and derivation of the dip positions. Although the analysis procedure was automated, for each FEL incident frequency we selected different areas in which to examine the dips and set different thresholds for minimum peak prominence and maximum peak width. In particular, looking at the s-SNOM images in Fig. 2 of the main text, we cannot detect the presence of a short ray for all FEL incident frequencies: for $\omega_{\mathrm{inc}} = [667, 697, 706]\,\mathrm{cm}^{-1}$, the short ray cannot be distinguished from the long ray in the upper half of the images. Therefore, in these cases we consider only two angular regions (instead of three). The dip positions, plotted as red circles in Fig. S1.3, were then averaged with a weighted mean using the peak prominence values as weights. This allowed us to determine the angle at which the rays propagate, which we then used to plot the red dashed lines in Fig. 2 of the main text.

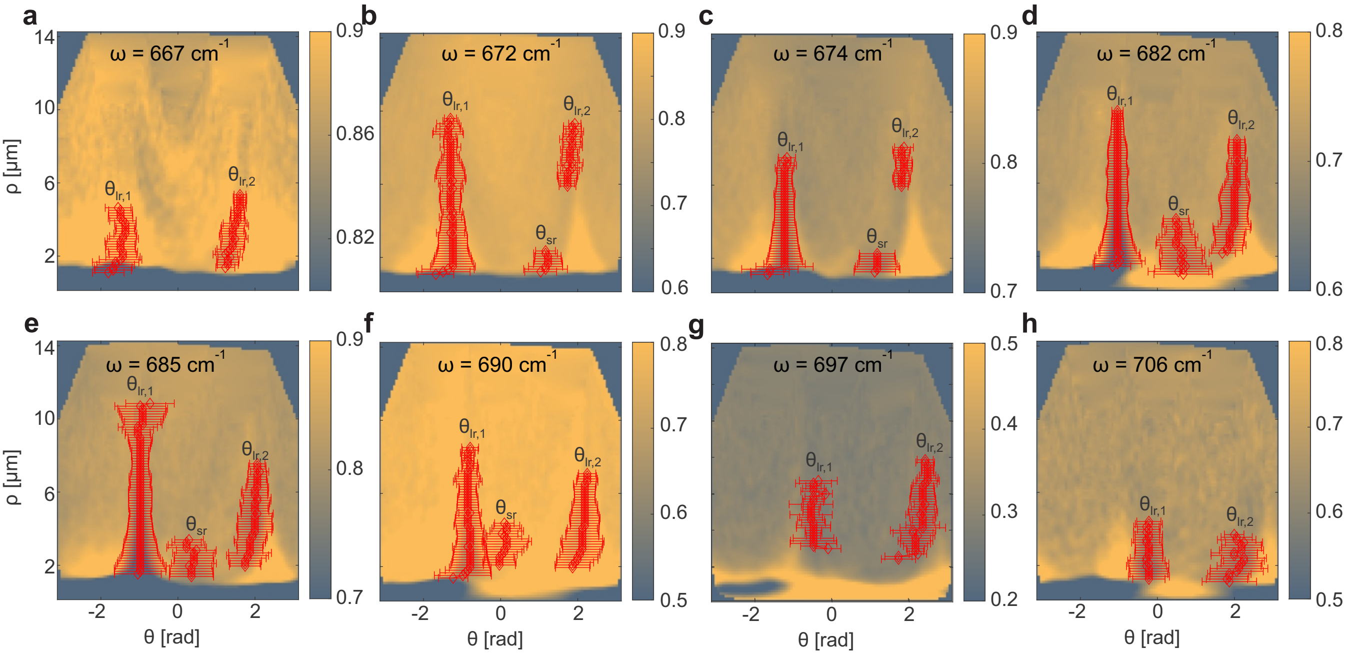

Figure S1.3: **Extension of analysis procedure to various frequencies**. (a-h) Near-field optical images converted to polar coordinates, overlaid with the dip positions of the longest segments for each angular region. Each panel corresponds to a different frequency of the FEL incident light. The error bars (red solid lines) correspond to the FWHM of the extracted dips.

S2 Experimental derivation of optical axis dispersion angle γ and opening angle α

This section elucidates the procedure followed to extract the experimental data shown in Fig. 4 of the main text. Experimentally, the angles γ_{exp} and α_{exp} were obtained after applying the whole procedure described in the previous sections S1-S3, and identifying two or three main rays in the s-SNOM images. Let us consider Fig. S1.3. In panels b-f we could recognize three main areas where the near-field optical signal has clear minima. We identified two long rays and one short ray, due to the strong optical anisotropy of the underlying crystal. For these incident frequencies, α_{exp} and γ_{exp} were calculated as follows:

$$\alpha_{\text{exp}} = \pi - |\theta_{\text{lr},1} - \theta_{\text{sr}}| \tag{S1}$$

$$\gamma_{\text{exp}} = \theta_{\text{lr},1} - \alpha_{\text{exp}}/2 \tag{S2}$$

In Fig. S1.3 a,g,h the short ray is not visible, since these frequencies are at the limits of the hyperbolic region, where we expect the arms of the hyperbola to converge. This transition can be observed in Fig. S1.3: while still absent in panel a, the short ray starts appearing in panel b from the long ray at $\theta_{\text{lr},2}$, from which it becomes increasingly separated at increasing frequencies (i.e., in the next panels), to finally merge again with the other long ray, $\theta_{\text{lr},1}$ in panel h. To account for this behaviour, we had to make two different cases for the two ends of the hyperbolic band: for panel a, $\alpha_{\text{exp}} = \pi - |\theta_{\text{lr},1} - \theta_{\text{lr},2}|$, and γ_{exp} was calculated as in eq. S1, while for panels g,h $\alpha_{\text{exp}} = |\theta_{\text{lr},1} - \theta_{\text{lr},2}|$ and $\gamma_{\text{exp}} = \theta_{\text{lr},1} - (\pi - \alpha_{\text{exp}}/2)$.

S3 Comparison with larger disc size

In this section we compare the main results with the data obtained using a disc with a larger diameter of 4 µm. As shown in a previous publication [5] and mentioned above (see section S1.2), the disc size plays an important role in determining the anisotropy in the polariton propagation. The directionality of the mode decreases with disc diameter: when launched from a 4 µm diameter disc, the polariton propagates with clearly visible tilted wave fronts instead of a ray-like behaviour. These wave fronts are useful in determining the polariton propagation direction, which is shown by the white solid lines in Fig. 2.

In Fig. S3.1, the raw data obtained with the 2 µm diameter disc (a) is compared with the near-field optical signal produced by a 4 µm diameter disc (b). While panel a shows a well-defined polariton propagating from the Au disc in a ray-like fashion, the pattern traced by the polariton in panel b appears more complex to interpret. The larger momentum modes launched by the smaller disc decay more rapidly, and the resulting patterns are free of artifacts (as displayed in Fig. S3.1 a). On the contrary, for the 4 µm diameter disc, we can distinguish two different fringe patterns:

1. The interference pattern with a shorter wavelength emerges at the air/^{18}O bGO film interface due to the lower asymmetry in propagation with respect to polaritons launched by smaller discs, which makes the wave fronts more pronounced, similar to previous observations in ^{16}O bGO bulk single crystals [5].

2. The other interference pattern component is due to the larger penetration depth of the low momentum modes into the film, which allows them to couple to the film/substrate interface.

Despite the complexity of the image, the shorter wavelength fringes (corresponding to the wave fronts of the hyperbolic shear polariton launched at the air/^{18}O bGO film interface) can be useful to determine the direction of propagation. In fact, we have chosen α_{exp} to be defined as in eq. S1, but we could just as well have defined this angle as $\pi - \alpha_{\text{exp}}$. In this case, γ_{exp} would be rotated by $\pi/2$. The comparison illustrated in Fig. S3.1 shows that the ambiguity can be overcome when the polariton wave fronts are taken into account.

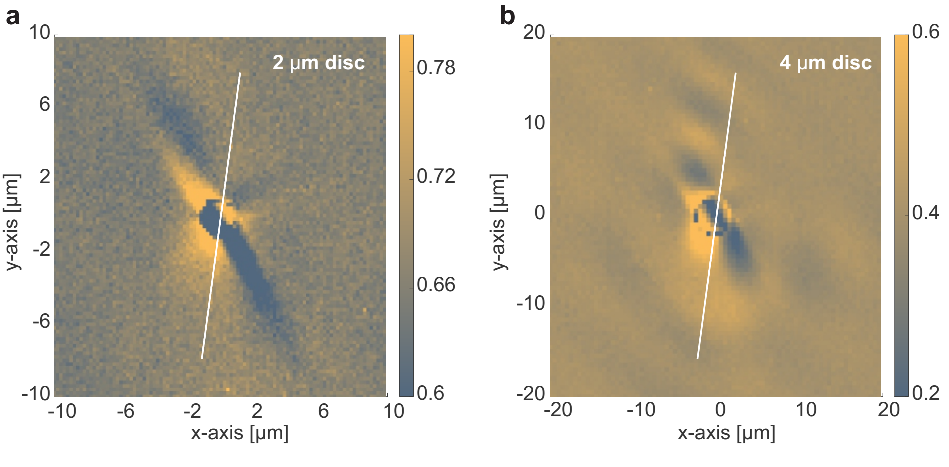

Figure S3.1: **Comparison between polaritons emitted by discs with different diameters**. (a-b) Second harmonic of the near-field optical signal for Au discs with diameters of 2 µm (a) and 4 µm (b) at an incident wavelength of 682 cm^{-1}. The solid white line indicates the polariton propagation direction derived from the analysis procedure discussed in the previous sections.

S4 s-SNOM data on ^{18}O bGO films of varying thickness

This section presents s-SNOM images of HShPs supported at the interface of ^{18}O bGO films of various thicknesses, which were epitaxially grown on a ^{16}O bGO substrate. These data are included for completeness, particularly to corroborate the choice of an intermediate thickness of 1.2 µm, see Fig. 2 of the main text. Our goal was to select the simplest system for studying the spectral shift induced by isotopic substitution, without further complications due e.g. to coupling with the substrate.

Fig. S4.1 shows s-SNOM images demodulated at the 2nd harmonic for 230 nm (a) and 1.8 µm (b) thick ^{18}O bGO films, recorded at an incident FEL frequency of $\sim$ 686 cm^{-1}. The polaritons are launched by Au discs with diameters of 4 µm and 2 µm, respectively. While both images bear some resemblance to the HShP in Fig. 2 e of the main text, at least on a qualitative level, they are both difficult to interpret for different reasons.

Fig. S4.1 a displays a s-SNOM image of HShPs at the interface of a 230 nm thick ^{18}O bGO film. In this case, the major issue is represented by the low signal-to-noise ratio, which compromises the image quality. Furthermore, in the thinner film, the interaction with the ^{16}O bGO substrate is non-trivial, and gives rise to effects that are harder to interpret. Fig. S4.1 b shows real-space propagation of HShPs at the interface of the 1.8 µm thick ^{18}O bGO film. The sample exhibits a poor surface quality, characterized by roughness and a high density of dust particles accumulated at the surface. The near-field signal of the antenna-launched polariton interferes quite significantly with the polaritons emitted by the dust particles, making this configuration not ideal for our purposes, as it renders the analysis even more complicated.

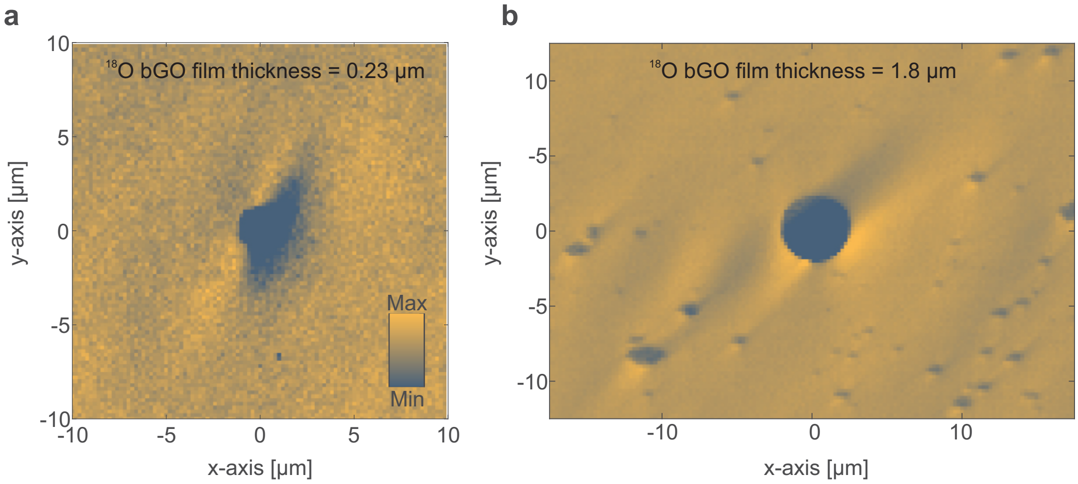

Figure S4.1: **Comparison between different ^{18}O bGO film thicknesses**. a,b) s-SNOM images of HShPs launched from 2 µm (a) and 4 µm (b) large Au antennae at the interface of 230 nm (a) and 1.8 µm (b) thick ^{18}O bGO films on a ^{16}O bGO substrate. The FEL incident frequency was set at $\omega \sim 686\,\mathrm{cm}^{-1}$.

S5 Study of hyperbolic shear polaritons in ^{16}O bGO

In this section, we study the propagation of hyperbolic shear polaritons (HShPs) on a ^{16}O bGO substrate. The experimental data points (blue squares) displayed in Figs. 4 b-c of the main text are extracted from the s-SNOM images in Fig. S5.1. To account for artifacts caused by the mixing of amplitude and phase channels resulting from the self-homodyne detection scheme employed in the experiment, we multiply our (intensity) images with the cosine of the phase of the lock-in amplifier [5, 8], as follows:

$$S_2 = -\text{O2A} \cdot \cos(\text{O2P}). \tag{S3}$$

The signal is demodulated at the second harmonic. The HShPs shown in Fig. S5.1 are launched by a Au disc with a 2 µm diameter. Furthermore, a circular mask with a radius of 1 µm centered on the disc has been applied to each s-SNOM image. The data are analyzed as described in detail in the previous sections of this SI. The red dashed lines are derived from the maxima in the distribution of the near-field signal for the long and the short ray. Note that in Fig. S5.1 b-e, we have selected only one quadrant for the long ray and one quadrant for the short ray. As we can see in the respective panels, the signal seems to undergo a phase flip in the opposite quadrant, causing the maxima to become minima. Therefore, the red dashed lines derived from the analysis overlap with the brightest regions of the images only in two quadrants. In Fig. S5.1 a, the short ray is not visible, because the polariton is canalized there due to the presence of a topological transition between a hyperbolic and an elliptical spectral regimes. The white solid line indicates the direction of the polariton propagation. Note that the angle γ_{exp} (as plotted in Fig. 4 of the main text) is defined with respect to the crystal axes as shown in Fig. S5.1 e.

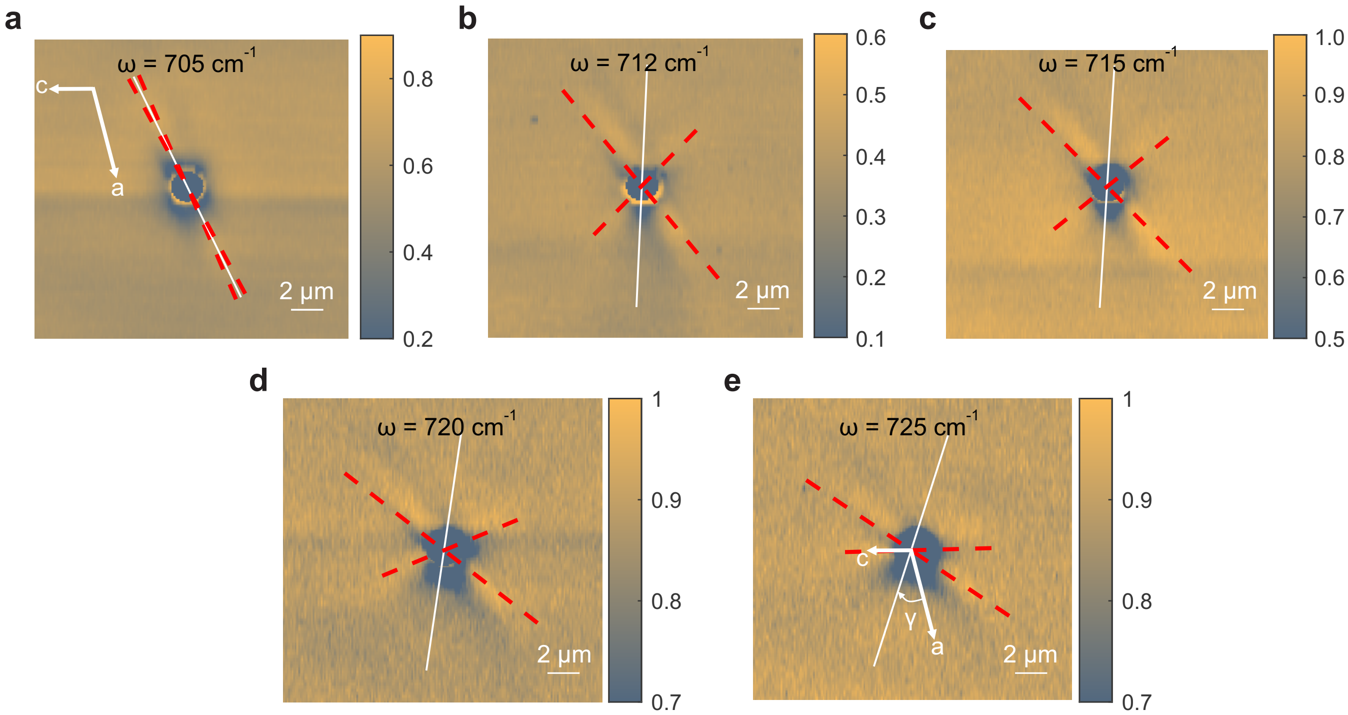

Figure S5.1: **Hyperbolic shear polariton real-space propagation in ^{16}O bGO**. (a-e) Second harmonic of the near-field optical signal for various wavenumbers in a ^{16}O bGO substrate. The polaritons are launched by Au discs with a diameter of 2 µm. The red dashed lines indicate the orientation of the polariton rays, while the white solid line illustrates the propagation direction. Panel e reports a sketch of the crystal axes and shows how the angle γ is defined relative to them.

S6 Experimental characterization of the dielectric permittivity tensor

The experimental parameters for the dielectric tensor components plotted in Fig. 3 a-c were derived from polarized reflectance measurements carried out using Fourier transform infrared (FTIR) spectroscopy. The measurements were performed (1) on a reference [010] ^{16}O bGO substrate, and (2) on the isotopically substituted ^{18}O bGO film homoepitaxially grown on a ^{16}O bGO substrate, utilizing a commercial FT-IR spectrometer (Bruker *Vertex 80V*) with a modified sample holder (A513/QA bench modified). The sample's reflectance was measured at a fixed angle of incidence of 15° for the ^{18}O bGO film, and at 50° for the reference ^{16}O bGO bulk crystal. The smaller angle of incidence was used for the ^{18}O bGO film to minimize the influence of the out-of-plane permittivity, which cannot be sufficiently accurately determined from a single crystal cut. The azimuthal orientation was varied from 0° to 180° at an increment of 5° for both isotopes. The detection scheme employed in the measurements consists of a deuterated L-alanine doped triglycine sulphate (DLaTGS) IR detector, a KRS5 polarizer, and a Si beam splitter.

The reflectance data for the respective materials are represented as reflectance contour maps in Fig. S6.1. These results show the polarization dependent/independent RBs [11] for the two materials: the polarization independent RBs exist for a given frequency at all azimuthal angles, while the polarization dependent ones only exists within a specific azimuthal range. The maps demonstrate how the isotopic substitution has modified the RBs within the IR spectra. However, it is important to note that in the data shown in Fig. S6.1 b both the ^{18}O bGO epilayer and the ^{16}O bGO substrate play a significant role.

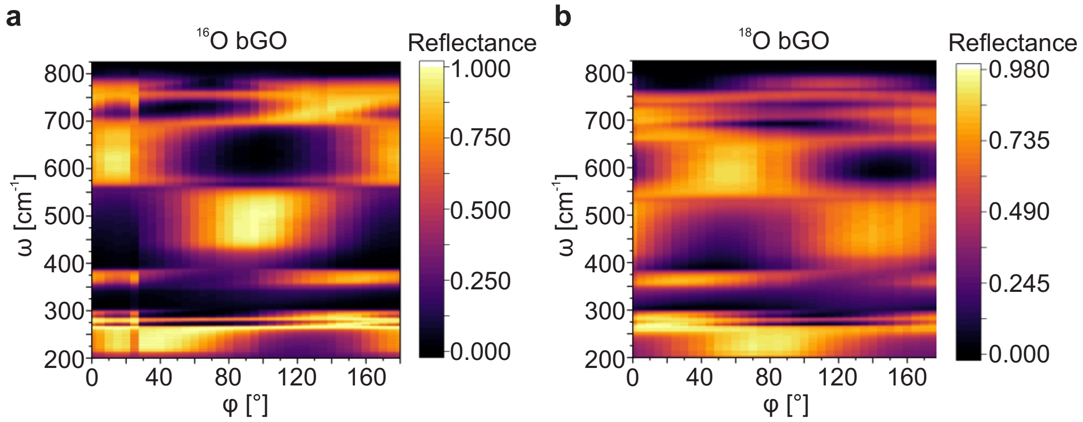

Figure S6.1: **FT-IR reflectance contour maps obtained from the raw data**. (a-b) FT-IR reflectance contour maps for the ^{16}O bGO bulk single crystal (a) and the ^{18}O bGO 1.2 μm thick film homoepitaxially grown on the ^{16}O bGO substrate (b).

The dielectric permittivity tensor was fitted using a multi-oscillator model, as first described in Ref. [10], using *WVASE* [1]. *WVASE* is a proprietary software for analyzing ellipsometry, transmission and reflectance data. The initial fitting was done for the ^{16}O bGO reflectance data using literature values from Ref. [10] of the IR active modes with B_u and A_u symmetry: the TO phonon frequencies $\omega_{TO,i}$, the oscillator strengths $\|\mathbf{S}_i\|$, the phonon angles δ_i, and the damping constants γ_i taken from the aforementioned reference served as initial parameters in the harmonic Lorentz oscillator model (see Methods in the main text) to fit the reflectance data. Before optimizing the parameters to fit the experimental data, we applied a ϕ Euler rotation of 65.5° in the xy-plane of the laboratory frame to match the literature data.

We could then proceed with the fitting for the ^{16}O bGO isotope in *WVASE*, starting from a single azimuthal angle, ϕ, and adding additional azimuthal angles piecewise to achieve a systematic fit. The parameters for the ^{18}O bGO were fitted following a similar approach, with the addition of a multilayer model accounting for the epitaxial

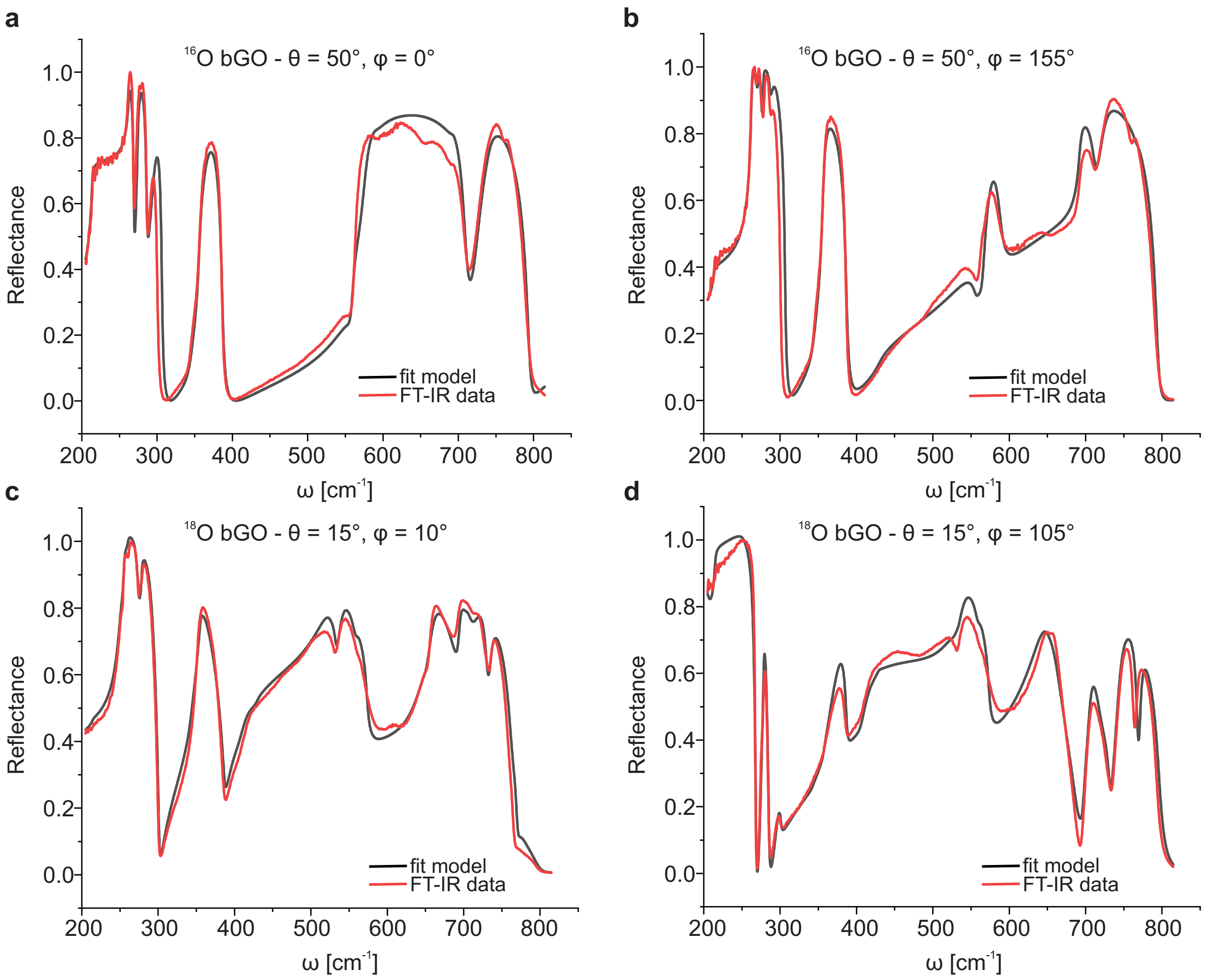

Figure S6.2: **Fitting procedure applied to derive the dielectric tensor from FT-IR measurements**. (a-b) Fitted (black lines) and raw experimental (red lines) plots for the ^{16}O bGO bulk single crystal for azimuthal angles, $\phi = 0°$ (a) and $\phi = 155°$ (b). (c-d) Fitted (black lines) and raw experimental (red lines) plots for the ^{18}O bGO epitaxial layer for azimuthal angles, $\phi = 10°$ (c) and $\phi = 105°$ (d). Note that the incidence angles θ chosen for the two isotopes are different, $\theta = 50°$ for ^{16}O bGO and $\theta = 15°$ for ^{18}O bGO.

structure - note that the sample consists of a ^{16}O bGO substrate and a 1.2 μm thick ^{18}O bGO epitaxial layer - in the *WVASE* software. While for the substrate layer of ^{16}O bGO we made use of the previous fitting results, a new model was required for the epitaxial layer of ^{18}O bGO. The initial parameters for the TO phonon frequencies in the case of the ^{18}O bGO epitaxial layer were taken from *ab initio* calculations, as shown in Table S1, while the remaining parameters were initially set to similar values as for the ^{16}O bGO isotope, and they were all fitted. With the multilayer model complete, the first parameter optimized was the necessary Euler rotation, followed by the optimized parameters of the ^{18}O layer. Finally, optimization is done with ^{16}O bGO parameters active along with ^{18}O bGO to ensure the contribution of generated reflectance spectra isn't only from the epi-layer. The fitting procedure uses the experimental data from ^{18}O bGO measurements for the listed parameters, including the out-of-plane phonon modes (with A_u symmetry).

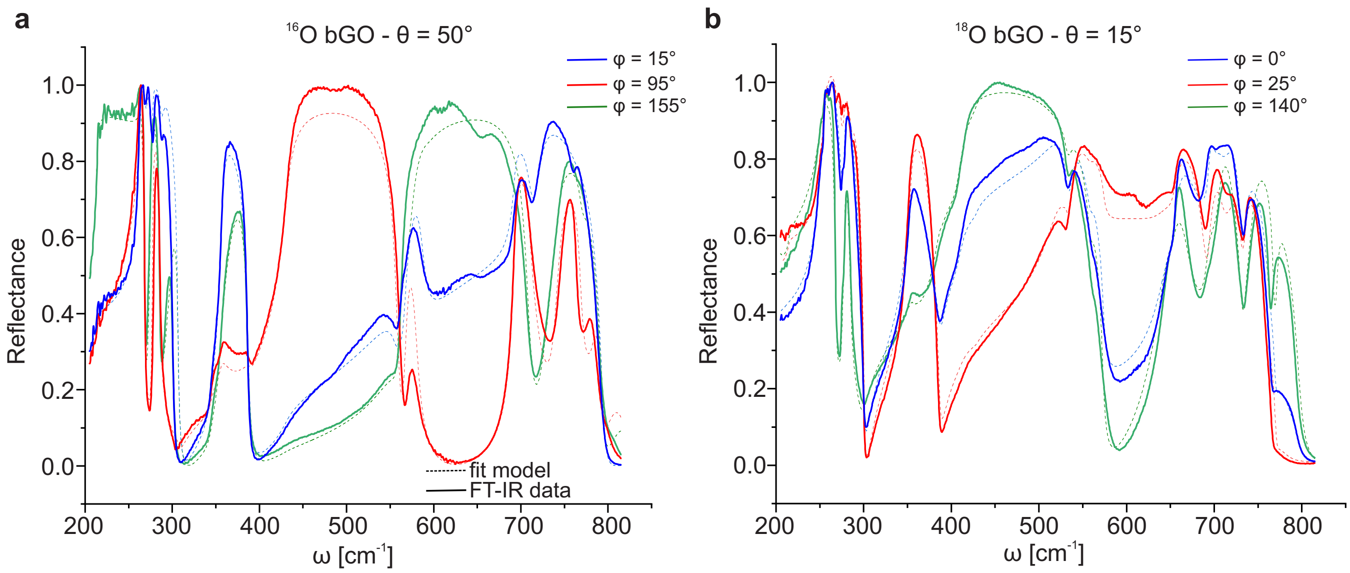

Figure S6.3: **Fitting procedure applied to derive the dielectric tensor from FT-IR measurements for various azimuthal angles**. (a) Fitted (dashed lines) and raw experimental (solid lines) plots for the ^{16}O bGO bulk single crystal for azimuthal angles, $\phi = 15°, 95°, 155°$ at a fixed incidence angle, $\theta = 50°$. (b) Fitted (dashed lines) and raw experimental (solid lines) plots for the ^{18}O bGO epitaxial layer for azimuthal angles, $\phi = 0°, 25°, 140°$ at a fixed incidence angle, $\theta = 15°$.

Fig. S6.2 shows a comparison of the experimental raw data (red lines) with the fitting results (black lines) for both bGO isotopes at two different azimuthal orientations. This demonstrates that the fitting model accurately reproduces the reflectance features of the IR active modes for both materials. The fitting parameters have reasonable uncertainty values and capture the prominent features of the reststrahlen bands. However, although the fitting can capture these features, it has significant limitations when it comes to replicating the experimental data for the tiniest details in the reststrahlen bands. This can largely be attributed to systematic errors in the measurements. First, all data sets have a cutoff at 200 cm^{-1}, that is, above the lowest frequency IR active mode (mode #8) with B_u symmetry, and rely on out-of-plane data from literature values only. Additionally, there are imperfections in the polarization state, angular spread and referencing inherent to reflection spectroscopy, all of which can introduce errors in measuring certain optical model parameters. Some of the systematic errors associated with these imperfections are discussed in the context of FT-IR microscopy in Ref. [7], but have not been systematically analyzed here. As such, while the model is sufficiently accurate for analysis of polariton data, it may not be fully representative of the true bGO optical constants. Azimuthal dispersion maps obtained from the fitting results are plotted in Figs. S6.4.

S7 Analytical derivation of the optical axis dispersion angle γ and of the opening angle α

Fig. 4 of the main text shows the optical axis and opening angle dispersion. The optical axis dispersion $\gamma(\omega)$ is measured as the angle between the coordinate system in which the permittivity tensor is measured (xyz) and the coordinate system that diagonalizes the real part of the permittivity tensor (mnz), as a function of frequency. At any given frequency, the basis vectors for the mnz coordinate system, in the xyz coordinates, are the eigenvectors $\hat{\mathbf{e}}$ of $\mathcal{R}(\varepsilon)$. The optical axis dispersion angle γ is then the angle between the positive x-axis of the xyz frame and the

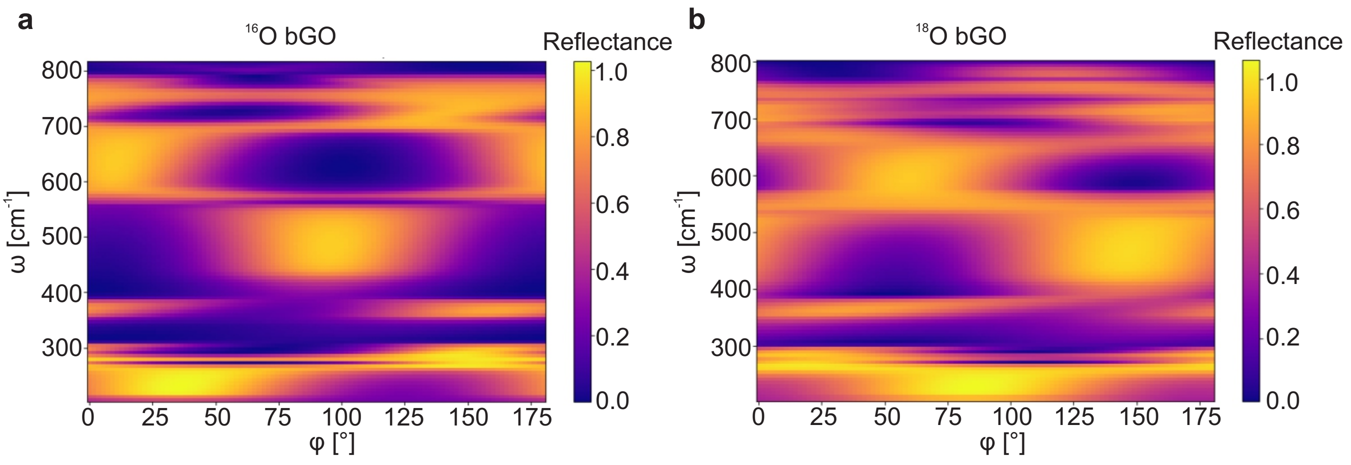

Figure S6.4: **FT-IR reflectance contour maps obtained from the *WVASE* fitting results**. (a-b) FT-IR reflectance contour maps obtained from the *WVASE* fitting results for the ^{16}O bGO bulk single crystal (a) and the ^{18}O bGO 1.2 μm thick film homoepitaxially grown on the ^{16}O bGO substrate (b).

Table S1: **Oxygen isotope effect on in-plane transverse optical (TO) phonons in bGO.** The experimental results were obtained from *WVASE* fitting of polarized FT-IR reflectance measurements performed at various azimuthal angles (see SI Section S6 [11]). All experimental values are reported with their fitting errors. The theoretical values are derived from *ab initio* calculations, as shown in the Methods (main text).

	$\mathbf{B_u}$ **mode**	Unit	**#1**	**#2**	**#3**	**#4**	**#5**	**#6**	**#7**	**#8**
Exp.	$\omega_{\mathrm{TO},18}$	cm^{-1}	703.6 ± 0.3	655.0 ± 0.4	538.1 ± 0.6	411.1 ± 0.5	352.9 ± 0.4	276.5 ± 0.4	254.9 ± 0.2	195 ± 2
Exp.	$\omega_{\mathrm{TO},16}$	cm^{-1}	742.5 ± 0.2	694.0 ± 0.1	567.2 ± 0.4	430.0 ± 0.3	356.2 ± 0.1	278.64 ± 0.08	260.5 ± 0.1	214.8 ± 0.1
Exp.	$\Delta\omega_{rel.}$	%	5.2	5.6	5.1	4.4	0.9	0.8	2.1	9.2
Th.	$\omega_{\mathrm{TO},18}$	cm^{-1}	696.76	647.02	545.89	409.47	350.01	274.37	244.18	184.14
Th.	$\omega_{\mathrm{TO},16}$	cm^{-1}	737.28	684.17	574.22	430.40	354.73	277.30	252.68	193.74
Th.	$\Delta\omega_{rel.}$	%	5.5	5.4	4.9	4.9	1.3	1.1	3.4	4.9
Exp.	$\lVert\mathbf{S}_{18}\rVert$	cm^{-1}	281 ± 3	432 ± 3	650 ± 8	772 ± 6	326 ± 5	118 ± 6	437 ± 5	460 ± 10
Exp.	$\lVert\mathbf{S}_{16}\rVert$	cm^{-1}	297 ± 2	406 ± 2	810 ± 4	772 ± 3	349 ± 2	153 ± 2	445 ± 2	519 ± 2
Th.	$\lVert\mathbf{S}_{18}\rVert$	cm^{-1}	436.54	578.37	1100.1	1059.5	401.22	90.09	571.25	639.42
Th.	$\lVert\mathbf{S}_{16}\rVert$	cm^{-1}	436.28	604.5	1148.9	1103.1	454.56	161.09	569.52	674.99
Exp.	δ_{18}	°	53 ± 4	5 ± 3	108 ± 4	22 ± 3	146 ± 3	3 ± 4	165 ± 3	82 ± 4
Exp.	δ_{16}	°	48 ± 2	5 ± 2	108 ± 2	25 ± 2	145 ± 2	7 ± 2	159 ± 2	81 ± 2
Th.	δ_{18}	°	72.67	25.82	127.79	44.45	156.25	12.42	182.03	105.26
Th.	δ_{16}	°	73.54	27.07	127.88	45.07	162.29	16.60	181.6	105.02
Exp.	γ_{18}	cm^{-1}	8.9 ± 0.3	13.5 ± 0.5	14.5 ± 0.6	16.2 ± 0.7	8.5 ± 0.5	6.0 ± 0.9	3.3 ± 0.3	1 ± 1
Exp.	γ_{16}	cm^{-1}	13.2 ± 0.4	3.9 ± 0.1	17.6 ± 0.3	11.5 ± 0.3	4.3 ± 0.1	1.72 ± 0.08	2.3 ± 0.1	2.3 ± 0.1
Th.	γ_{18}	cm^{-1}	11.55	5.33	13.48	10.88	8.34	1.78	1.37	1.15
Th.	γ_{16}	cm^{-1}	11.55	5.33	13.48	10.88	3.83	1.78	1.37	0.97

eigenvector $\hat{\mathbf{e}}_{\mathrm{m}}$.

In the following, we will compare two different procedures for deriving the angle γ, in order to highlight the novelty of our approach with respect to previous studies [5, 9]. The usual derivation method consists in applying a rotation to the original ε-tensor in Cartesian coordinates, $R(\gamma)\,\mathcal{R}(\varepsilon_{\mathrm{xyz}})\,R^{-1}(\gamma) = \mathcal{R}(\varepsilon_{\mathrm{mnz}})$. Setting the off-diagonal terms of $\varepsilon_{\mathrm{mnz}}$ to zero then leads to the equation:

Table S2: **Electronic contributions to the dielectric tensor for the ^{16}O and ^{18}O bGO isotopes.**

	$\varepsilon_{\infty,\mathbf{xx}}$	$\varepsilon_{\infty,\mathbf{yy}}$	$\varepsilon_{\infty,\mathbf{zz}}$
Exp. ^{18}	3.69 ± 0.05	3.18 ± 0.06	4 ± 2
Exp. ^{16}	3.78 ± 0.03	3.59 ± 0.03	4.042 ± 0.002
Th. ^{18}	3.883	3.995	4.000
Th. ^{16}	3.883	3.995	4.000

$$\begin{aligned}(\cos^2(\gamma) - \sin^2(\gamma))\mathcal{R}(\varepsilon_{\rm xy}) + \cos(\gamma)\sin(\gamma)(\mathcal{R}(\varepsilon_{\rm yy}) - \mathcal{R}(\varepsilon_{\rm xx})) &= \\ = \cos(2\gamma)\mathcal{R}(\varepsilon_{\rm xy}) + \frac{1}{2}\sin(2\gamma)(\mathcal{R}(\varepsilon_{\rm yy}) - \mathcal{R}(\varepsilon_{\rm xx})) &= 0\end{aligned} \tag{S4}$$

Eq. S4 is solved by defining γ as follows,

$$\gamma = \frac{1}{2}\,\mathrm{atan}\left(2\,\frac{\mathcal{R}(\varepsilon_{xy})}{\mathcal{R}(\varepsilon_{xx}) - \mathcal{R}(\varepsilon_{yy})}\right). \tag{S5}$$

We notice here the existence of a 90° phase ambiguity in the definition of γ, due to the fact that eq. S4 may be equally solved by $\gamma' = \gamma + \frac{\pi}{2}$. When derived with this procedure, γ is only defined within a restricted domain of existence, i.e., between $-\pi/4$ to $+\pi/4$, which does not correspond to the periodicity of the physical optical axes dispersion. Furthermore, another problem is that this method introduces discontinuities at the TO frequencies, which result in sudden phase jumps occurring as a consequence of the arctan function going from $-\pi/2$ to $+\pi/2$ when its argument oscillates between $-\infty$ to $+\infty$.

The new procedure, developed in this work, tackles and solves these issues. Hereby, we present a thorough description of it. Instead of using eq. S5, we employ the equation that is given in the main text (eq. 1), which relies on the eigenvectors of the dielectric tensor in cartesian coordinates, $\varepsilon_{\rm xyz}$. For this method, the first step is to make a consistent choice about which of the two eigenvectors of $\mathcal{R}(\boldsymbol{\varepsilon})$ that span the monoclinic plane is chosen as the reference to which γ is measured (see Fig S7.1). Moreover, both $\hat{\mathbf{e}}_{\rm m}$ and $-\hat{\mathbf{e}}_{\rm m}$ are eigenvectors of $\mathcal{R}(\boldsymbol{\varepsilon})$, and a similarly consistent sign convention has to be kept across all frequencies at which γ is computed. Otherwise, 90-degree discontinuities in γ are introduced (e.g., Fig. S7.1 c and Fig. S7.2 c), which reflect an inconsistency in the choice of the basis vectors for the mnz coordinate system and not a physically meaningful rotation in the optical axis. The consistency is maintained by aligning the eigenvectors at frequency $\omega_{\rm i}$ with those obtained at the previous frequency $\omega_{\text{i-1}}$. The alignment is done maximizing $\hat{\mathbf{e}}_{\rm m/n}(\omega_{\rm i}) \cdot \hat{\mathbf{e}}_{\rm m/n}(\omega_{\text{i-1}})$ by permuting which eigenvector at $\omega_{\rm i}$ is labeled m and which - n and by inverting them, as appropriate.

The above-described "eigenvector alignment" procedure for determining $\gamma(\omega)$ is not foolproof, because the optical losses (Lorentzian damping constant $\gamma_{\rm i}$ of eq. 3, main text) smooth the transitions of $\boldsymbol{\varepsilon}$ across the resonance frequencies $\omega_{\rm TO}$. This causes the $\hat{\mathbf{e}}_{\rm m}$ to continuously rotate by ~90 degrees across $\omega_{\rm TO}$ resulting in a smooth, but artificial near-90-degree shifts in $\gamma(\omega)$. A convenient strategy is to exclude $\boldsymbol{\varepsilon}$ values about 1-2 cm^{-1} of either side of $\omega_{\rm TO}$. In most cases, the resulting eigenvectors of the last $\mathcal{R}(\boldsymbol{\varepsilon})$ value before and the first value after $\omega_{\rm TO}$ are then aligned and chosen consistently. In the few remaining cases, the frequency range where the $\hat{\mathbf{e}}_{\rm m}$ was chosen inconsistently with respect to those of the frequencies before, the resulting 90-degree shifts have to be accounted by on a case-by-case basis, for example, by shifting the corresponding $\gamma(\omega)$ region by an appropriate 90-degree-multiple shift. Since the origin of the smooth yet artificial $\hat{\mathbf{e}}_{\rm m}$ rotation through $\omega_{\rm TO}$ are the optical losses, the genuine cases of rapid $\gamma(\omega)$ rotation from the artificial ones may be distinguished by setting the Lorentzian broadening $\gamma_{\rm i}$ to an arbitrary small value, see Fig. S7.2. With this approach in place, $\gamma(\omega)$ is a 360-degree periodic and mostly rotates clockwise with frequency, with only a few rapid rotations around, e.g., at $\omega_{\rm TO} \approx 276$cm^{-1} - see Fig. S7.3.

The angle γ presented above can then be used to diagonalize $\varepsilon_{\rm xyz}$. The components of the ε-tensor in the rotated frame ($\varepsilon_{\rm mnz}$) are displayed in Fig. S7.4 for a restricted spectral range relevant for our experiment, i.e., 640-750 cm^{-1}.

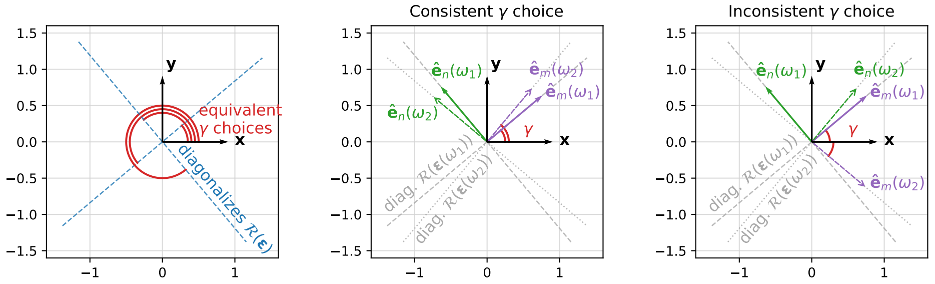

Figure S7.1: **The effect of inconsistent reference axis $\hat{\mathbf{e}}_m$ choice on the optical axis angle γ.** (a) Multiple values of γ, differing by multiples of 90 degrees, can describe the same mnz coordinate system that diagonalizes the permittivity tensor. As the mnz coordinate system rotates with frequency, the choice of the reference axis $\hat{\mathbf{e}}_m$ to which γ is measured needs to be consistent. (b) A consistent choice of $\hat{\mathbf{e}}_{\mathrm{m}}$ between frequencies ω_1 and ω_2. The change in γ reflects the fact that the coordinate system that diagonalizes $\mathcal{R}(\varepsilon)$ has rotated by 10 degrees counter-clockwise. (c) An inconsistent choice of the mnz coordinate basis vectors $\hat{\mathbf{e}}_{\mathrm{m}}$ and $\hat{\mathbf{e}}_{\mathrm{n}}$ between frequencies ω_1 and ω_2. The change in gamma from 40 degrees to -40 degrees reflects the inconsistency of the axis/basis vector to which γ has been measured, rather than the rotation of the optical axis by 80 degrees counter-clockwise.

The curves were derived from FT-IR and DFT data, see blue and orange curves, respectively. Note that the rotation applied can only diagonalize the real part of $\varepsilon_{\mathrm{xyz}}$. A full diagonalization of both the real and imaginary parts of the complex-valued dielectric tensor would require some other kind of transformation (involving non-hermitian operators, $\hat{O} \neq \hat{O}^{\dagger}$). Thus, in our case (since ε is a complex-valued tensor), the imaginary parts of the off-diagonal elements do not disappear and are plotted in Fig. S7.4 d,h for both bGO isotopes. For a more in-depth explanation, see Ref. [9]. The light-blue and grey shaded areas in Figs. S7.4 b-d and f-h indicate the spectral ranges where different types of polaritons are supported: the two colors correspond to hyperbolic and elliptical regions, respectively. Note that the polariton bands were classified based on the zero crossings calculated from the FT-IR permittivity data. In the left panels relative to the ^{16}O bGO isotope, we observe two elliptical regions of similar widths, i.e., spectral ranges where the real parts of both in-plane diagonal elements of the $\varepsilon_{\mathrm{mnz}}$-tensor are negative. The first elliptical region for the ^{18}O bGO isotope, shown in Fig. S7.4 g at $\omega \approx 655\,\mathrm{cm}^{-1}$, is much narrower, which is due to a combination of two main factors: 1) the large damping constant γ_{18} of the $\mathrm{B_u}$ mode #2 for the ^{18}O isotope (by a factor of 3.5 larger than the corresponding value for the ^{16}O isotope); 2) the smaller damping constant γ_{18} of the $\mathrm{B_u}$ mode #1 compared to the corresponding value for the ^{16}O isotope.

The angle α is derived geometrically from the ratio of the in-plane k-vector components in the newly defined reference frame, i.e., $\mathrm{k_{mm}}$ and $\mathrm{k_{nn}}$. Note that, for in-plane hyperbolic biaxial crystals, this ratio is related to the permittivity components by the equation $\frac{k_{\mathrm{mm}}}{k_{\mathrm{nn}}} = \sqrt{-\frac{\mathcal{R}(\varepsilon_{\mathrm{nn}})}{\mathcal{R}(\varepsilon_{\mathrm{mm}})}}$, as demonstrated in Ref. [3]. To summarize, α is defined by the following expression:

$$\alpha = 2\,\mathrm{atan}\left(\frac{k_{\mathrm{mm}}}{k_{\mathrm{nn}}}\right) = 2\,\mathrm{atan}\left(\sqrt{-\frac{\mathcal{R}(\varepsilon_{\mathrm{nn}})}{\mathcal{R}(\varepsilon_{\mathrm{mm}})}}\right). \tag{S6}$$

S8 Error estimation for s-SNOM and FT-IR data

This section is aimed at explaining the error estimation for both s-SNOM and far-field IR-reflectance data plotted in Fig. 4 of the main text. The vertical error bars have been calculated from the uncertainties on the weighted average

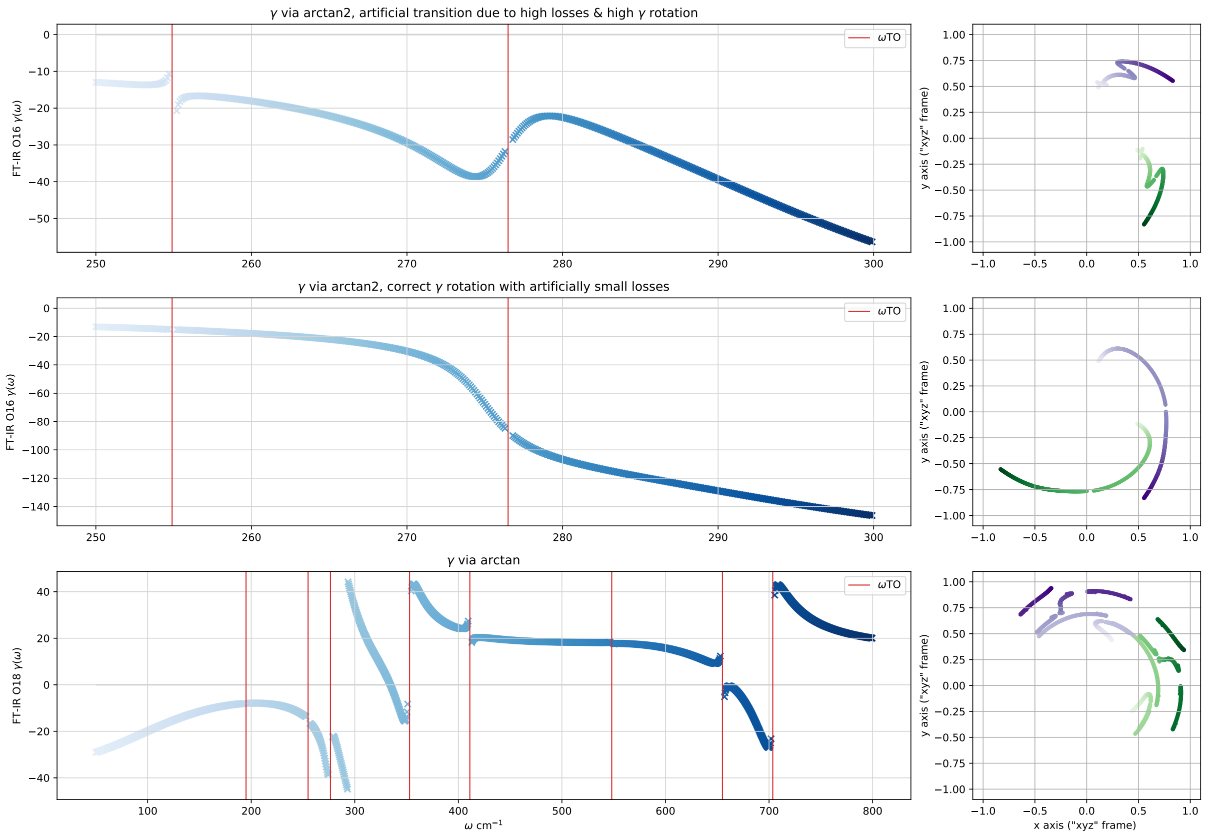

Figure S7.2: **Examples of challenges in computing $\gamma(\omega)$ in a consistent way across the full frequency range**. (a-b) $\gamma(\omega)$ and $\hat{\mathbf{e}}_{\mathrm{m}}$ & $\hat{\mathbf{e}}_{\mathrm{n}}$ rotation with frequency, near mode #6 ω_{TO} for the FT-IR O18 data. The permittivity was computed either with the experimentally measured Lorentzian broadening γ_i (a) or with an arbitrarily small value (b). The fact that γ shifts downwards (rotates clock-wise) through ω_{TO} in the permittivity model with artificially small broadening suggests that the wiggle in γ computed with experimentally measured broadening is a failure of "eigenvector alignment" procedure of computing γ rather than a physically meaningful rotation of the optical axis. In the rest of the analysis, the $\gamma(\omega)$ values to the right of $\omega_{\mathrm{TO}} \approx 273\mathrm{cm}^{-1}$ in panel (a) have been shifted down by 90 degrees to be consistent with the results in panel (b). (c) $\gamma(\omega)$ using eq. S5. Without further adjustment, γ values are defined between ± 90 degrees, resulting in discontinuous rotation of $\hat{\mathbf{e}}$.

of the long and short ray's angles ($\theta_{\mathrm{lr},1/2}$, θ_{sr}) as follows:

$$(\sigma_{\bar{x}})_{wtd} = \sqrt{\frac{\sum_{i=1}^{n}(w_i(x_i - \bar{x}_{wtd})^2)}{\sum_{i=1}^{n} w_i} \frac{1}{n_{eff} - 1}} \tag{S7}$$

where n_{eff} is the effective number of degrees of freedom, $n_{eff} = \frac{(\sum_{i=1}^{n} w_i)^2}{\sum_{i=1}^{n}(w_i^2)}$. In this way, the standard deviation from the weighted average is taken into account and the effective number of measurements depends on their weights. The horizontal error bars have been set to a length of 4 cm^{-1}, assuming a 0.5% FWHM of the incident FEL frequency.

We also included the min/max estimate for oscillator strength and damping (1. highest oscillator strength and lowest damping, 2. lowest oscillator strength and highest damping) from the *WVASE* fits to the IR-reflectance maps,

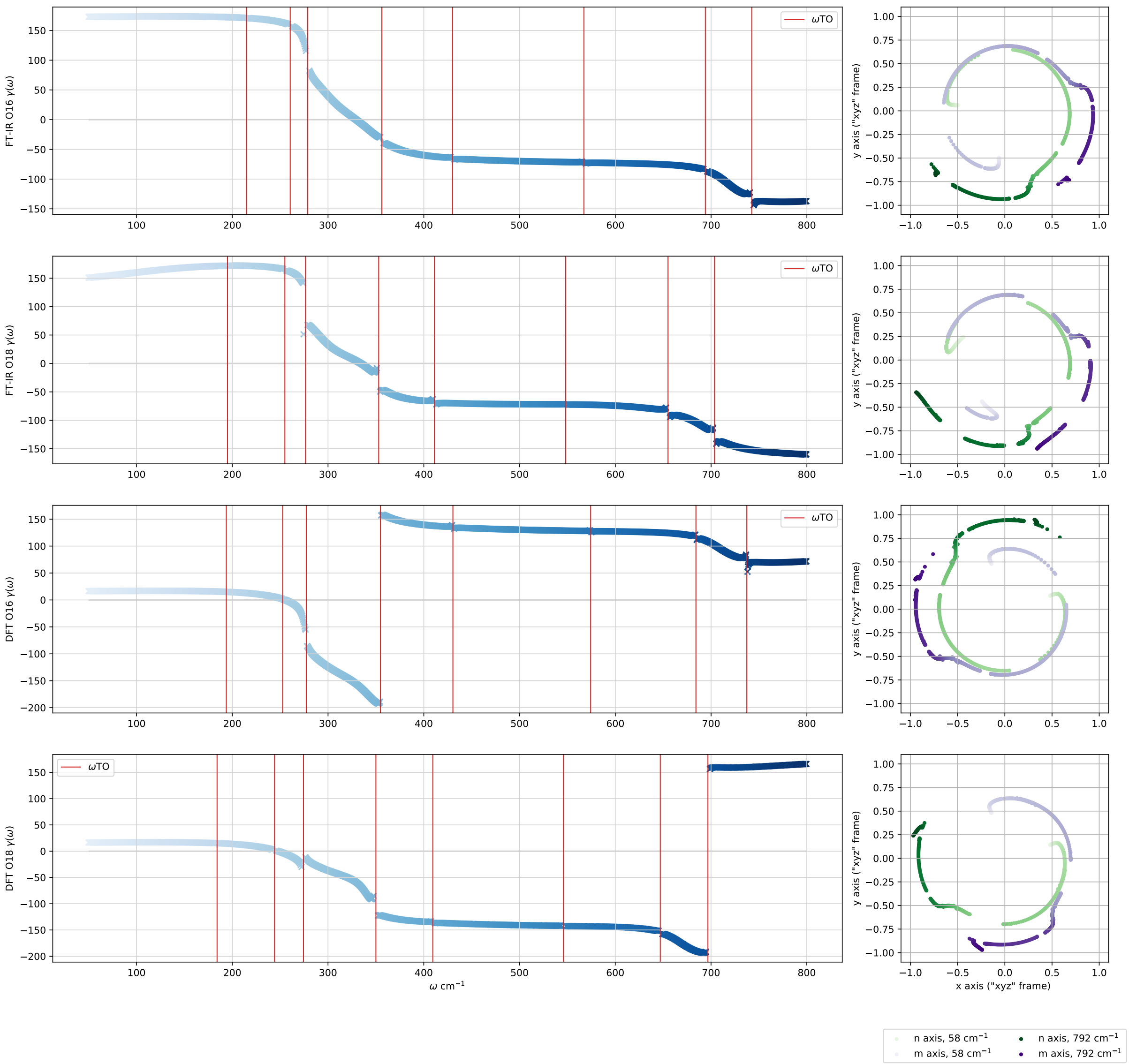

Figure S7.3: **Optical axis rotation with frequency for FT IR (a-b) and DFT (c-d) data, O16 (a, c) and O18 (b, d) bGO isotopologues.**

in addition to a ± 0.5 cm^{-1} horizontal uncertainty to account for the errors on the TO phonon frequencies.

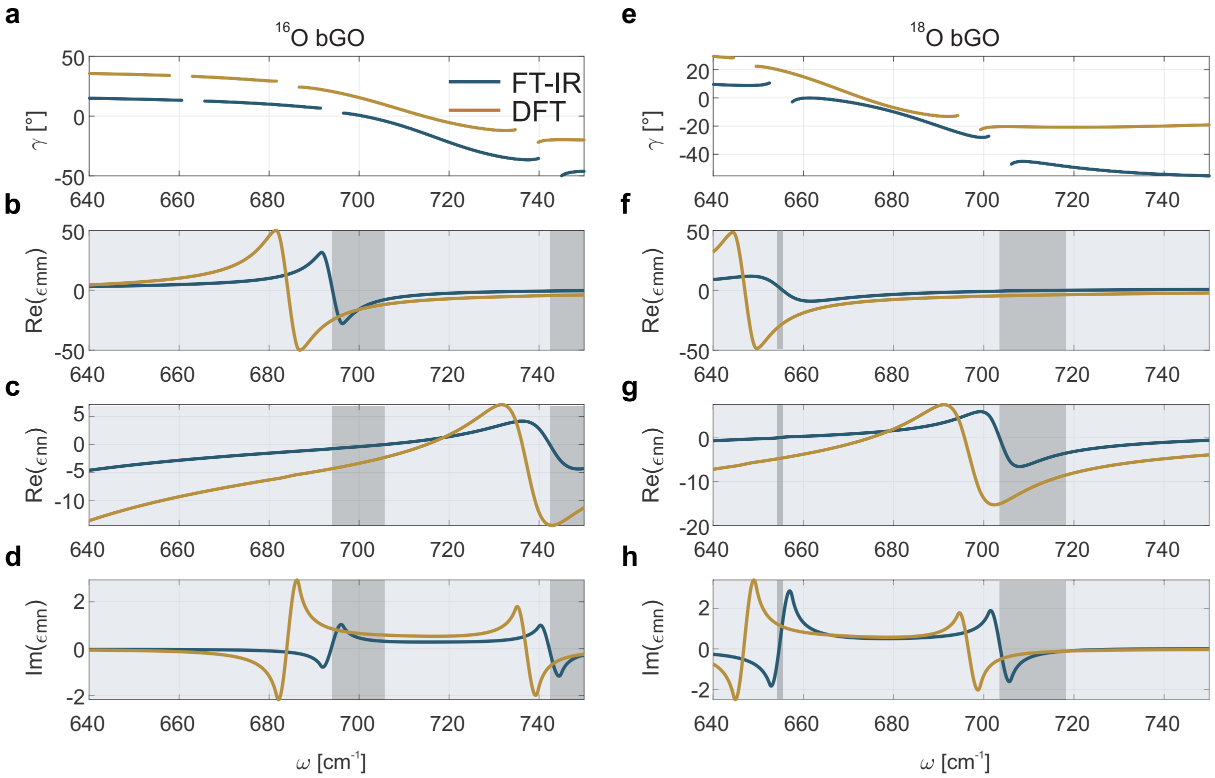

Figure S7.4: **Rotated ε-tensor components for the ^{16}O and ^{18}O bGO isotopes extracted from FT-IR (blue) and DFT (orange) data**. (a,e) Frequency dependence of the optical axis dispersion angle γ for the two bGO isotopes. (b,f,c,g) Real parts of the rotated ε-tensor diagonal elements for the two bGO isotopes. The shaded areas correspond to the spectral ranges where different types of phonon polaritons are supported: light blue indicates hyperbolic regions, whilst dark grey identifies elliptical polaritonic ranges. (d,h) Imaginary part of the off-diagonal element $\varepsilon_{\mathrm{mn}}$.

S9 Crystal orientation

X-ray diffraction (XRD) on a four-circle diffractometer (Malvern Panalytical X'Pert MRD) was performed on the ^{18}bGO to determine its in-plane crystallographic orientations. To probe the in-plane component for the investigated (010) homolayer, Φ-scans were performed for the (-112) and (221) planes, which give information for the [001] and [102] orientations, respectively. The spacing of the reflexes was confirmed by the stereographic projection generated using *WinWulff* and labeled as reported in Fig. S9.1(a). It is important to note that due to the two-fold symmetry of the (010) surface, only the [001] direction can be highlighted but not its absolute value (+ or -c), regardless, for the same reason, this does not play a relevant role for the current investigation. To confirm the crystallographic orientation extracted from the XRD measurements, additional AFM measurements were performed (Fig. S9.1(b)); in fact, (010) oriented homoepitaxial layers usually show peculiar features on its growth surface, i.e., the elongated features which are mostly orthogonal to the [001], trace of the anisotropic surface ad-atom diffusion during growth [6, 12].

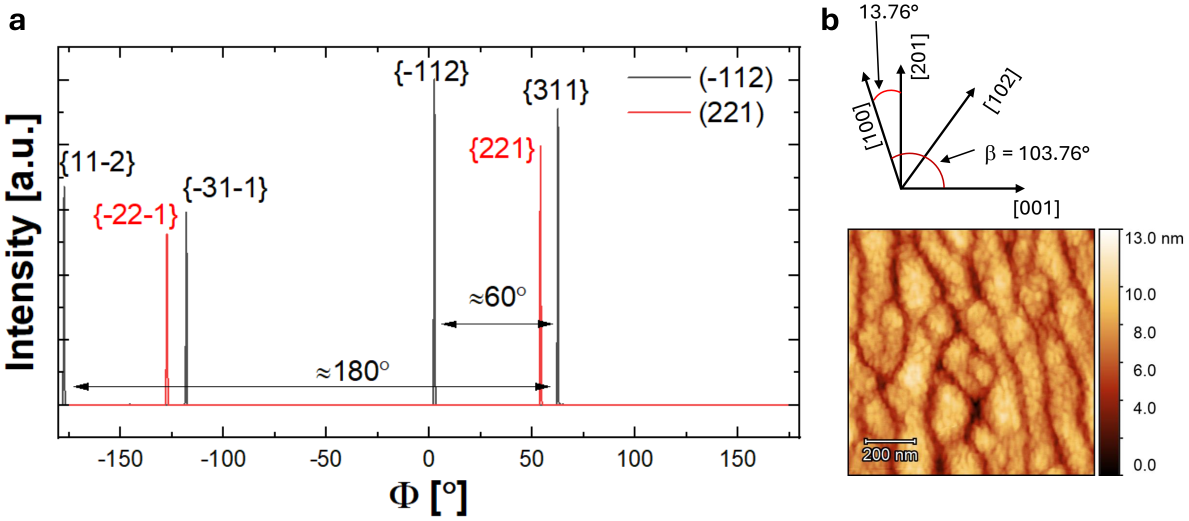

Figure S9.1: **Determination of the bGO In-plane orientations.** (a) Φ-scans for the (-112) and (221) planes to determine the [001] in-plane direction of the ^{18}bGO sample. (b) AFM image of the bGO with the reference crystallographic directions as determined by XRD.

S10 Penetration depth estimation of the polariton evanescent wave inside the ^{18}O bGO epitaxial film

To estimate the penetration depth of the SPhPs in our sample, we use the following formula:

$$d_{\pm} = \frac{1}{\kappa_{\pm}} \tag{S8}$$

where $\kappa_{\pm}$ is ik_z, i.e., the z-component of the k-vector. In the presence of evanescence, the decay constants $\kappa_{\pm}$ are always real and positive. The notation ± indicates the specific evanescent wave under consideration, with each wave decaying into one of the two half-spaces, filled with bGO (+) and air (-), respectively. By applying the boundary

conditions on the wave vector components parallel to the interface, we utilize the dispersion relation of bulk phonon polaritons in isotropic materials to derive the following expression for $\kappa_\pm$:

$$\kappa_\pm = k_0\sqrt{k_{x,0}^2 - \varepsilon_\pm} \tag{S9}$$

Considering incident light with a wavenumber, $\omega_0 = 700\,\text{cm}^{-1}$, we find the corresponding wavevector magnitude to be $k_0 = 2\pi\omega_0 = 0.44\,\mu\text{m}^{-1}$. On the bGO side, we have a large negative ε_-, due to the material being in the reststrahlen band, specifically, $\varepsilon_{zz} \approx -4$ along the z-direction. This results in a penetration depth on the bGO side of approximately $d_{\text{bGO}} = 0.23\,\mu\text{m}$. In contrast, the skin depth, which is relevant for electric fields, is calculated to be $0.46\,\mu\text{m}$. This difference of a factor of 2 between the two values arises because the penetration depth is calculated for intensities, while the skin depth pertains to electric fields. When considering an Au disc with a thickness of $4\,\mu\text{m}$, the skin depth into the bGO half-space is estimated to be $0.84\,\mu\text{m}$, assuming $k_{x,0} \approx 5$.

References

[1] WVASE - ellipsometric analysis program. `https://www.jawoollam.com/ellipsometry-software/wvase`. Accessed: 2025-05-16.

[2] J Álvarez-Cuervo, M Obst, S Dixit, AI F. Tresguerres-Mata, C Lanza, E Terán-García, LF Álvarez-Tomillo, K Diaz-Granados, et al. Unidirectional ray polaritons in twisted asymmetric stacks. *Nature communications*, 15(1):9042, 2024.

[3] Gonzalo Álvarez-Pérez, Kirill V Voronin, Valentyn S Volkov, Pablo Alonso-González, and Alexey Y Nikitin. Analytical approximations for the dispersion of electromagnetic modes in slabs of biaxial crystals. *Physical Review B*, 100(23):235408, 2019.

[4] Xinzhong Chen, Debo Hu, Ryan Mescall, Guanjun You, DN Basov, Qing Dai, and Mengkun Liu. Modern scattering-type scanning near-field optical microscopy for advanced material research. *Advanced Materials*, 31(24):1804774, 2019.

[5] Joseph Matson, Sören Wasserroth, Xiang Ni, Maximilian Obst, Katja Diaz-Granados, Giulia Carini, Enrico Maria Renzi, Emanuele Galiffi, Thomas G Folland, Lukas M Eng, et al. Controlling the propagation asymmetry of hyperbolic shear polaritons in beta-gallium oxide. *Nature communications*, 14(1):5240, 2023.

[6] P Mazzolini and O Bierwagen. Towards smooth (010) β-ga_2o_3 films homoepitaxially grown by plasma assisted molecular beam epitaxy: the impact of substrate offcut and metal-to-oxygen flux ratio. *Journal of Physics D: Applied Physics*, 53(35):354003, jun 2020.

[7] Siddharth Nandanwar, Aditya Desai, S Esfidani, Tristan McMillan, Eli Janzen, James H Edgar, and Thomas G Folland. Determining the optical and polaritonic properties of isotopically pure hbn using cryogenic ftir micro-spectroscopy. *Applied Physics Letters*, 126(1), 2025.

[8] Maximilian Obst, Tobias Nörenberg, Gonzalo Álvarez-Pérez, Thales VAG de Oliveira, Javier Taboada-Gutiérrez, Flávio H Feres, Felix G Kaps, Osama Hatem, Andrei Luferau, Alexey Y Nikitin, et al. Terahertz twistoptics–engineering canalized phonon polaritons. *ACS nano*, 17(19):19313–19322, 2023.

[9] Nikolai C Passler, Xiang Ni, Guangwei Hu, Joseph R Matson, Giulia Carini, Martin Wolf, Mathias Schubert, Andrea Alù, Joshua D Caldwell, Thomas G Folland, et al. Hyperbolic shear polaritons in low-symmetry crystals. *Nature*, 602(7898):595–600, 2022.

[10] Mathias Schubert, Rafal Korlacki, Sean Knight, Tino Hofmann, Stefan Schöche, Vanya Darakchieva, Erik Janzén, Bo Monemar, Daniela Gogova, Q-T Thieu, et al. Anisotropy, phonon modes, and free charge carrier parameters in monoclinic β-gallium oxide single crystals. *Physical Review B*, 93(12):125209, 2016.

[11] Mathias Schubert, Alyssa Mock, Rafał Korlacki, and Vanya Darakchieva. Phonon order and reststrahlen bands of polar vibrations in crystals with monoclinic symmetry. *Physical Review B*, 99(4):041201, 2019.

[12] Zhuoqun Wen, Kamruzzaman Khan, Xin Zhai, and Elaheh Ahmadi. Si doping of β-ga$_2$o$_3$ by disilane via hybrid plasma-assisted molecular beam epitaxy. *Applied Physics Letters*, 122(8):082101, 02 2023.
